# Label-free microscopy enables high-throughput identification of genes controlling biofilm development

**DOI:** 10.1128/mbio.00448-26

**Published:** 2026-05-13

**Authors:** M. R. Pratyush, Jojo A. Prentice, Rory A. Eutsey, Irina V. Mikheyeva, N. Luisa Hiller, Andrew A. Bridges

**Affiliations:** 1 Department of Biological Sciences, Carnegie Mellon University 171620, Pittsburgh, Pennsylvania, USA; Indiana University Bloomington, Bloomington, Indiana, USA

**Keywords:** biofilms, video microscopy, genetics, *Streptococcus pneumoniae*, *Vibrio cholerae*

## Abstract

Biofilms are structured microbial communities that thrive on diverse surfaces in natural, industrial, and host environments. The biofilm lifestyle underpins microbial survival, shapes ecosystem function, and drives persistent infections; yet, for many microbes, the molecular determinants of biofilm development remain poorly defined. Here, we introduce “label-free analysis of biofilms” (LFAB), an imaging method that integrates time-lapse, low-magnification brightfield microscopy with regional optical density measurements to quantify biofilm biomass. Unlike conventional assays, LFAB enables real-time, non-perturbative, and high-throughput monitoring of biofilm dynamics. We validated LFAB across diverse microbial species and observed a strong correlation with traditional biofilm quantification methods. Applying LFAB to *Streptococcus pneumoniae*, a major human pathogen whose biofilm lifecycle underpins colonization and infection, we uncovered reproducible patterns of microcolony biofilm expansion and growth. LFAB-enabled screening of a transposon mutant library revealed that biofilm formation in *S. pneumoniae* is shaped by genes spanning carbohydrate metabolism, cell wall synthesis, adhesion, and surface interactions. Further analysis identified choline-binding protein A (CbpA) and its associated two-component regulator, as well as the peptidoglycan hydrolase LytB, as key drivers of microcolony biofilm dynamics. Together, these findings establish LFAB as a broadly applicable platform for dissecting biofilm biology and reveal new regulators of biofilm development in a clinically important pathogen.

**IMPORTANCE:**

Biofilms are structured communities of microorganisms that attach to surfaces and persist within a self-produced matrix. The biofilm lifestyle underlies microbial survival in nature, contributes to industrial biofouling, and drives many chronic infections. Despite the importance of biofilms, high-throughput measurements of biofilm growth dynamics are challenging using existing tools, which are often disruptive or are not scalable. To overcome this limitation, we developed “label-free analysis of biofilms” (LFAB), a brightfield-based imaging platform that enables real-time, non-perturbative, and scalable quantification of biofilm biomass. LFAB is broadly applicable across species and correlates strongly with traditional assays. Applying LFAB to *Streptococcus pneumoniae*, a major human pathogen, we performed a mutagenesis screen, uncovering new genetic regulators of biofilm formation in this organism. These findings advance understanding of *S. pneumoniae* pathogenesis and establish LFAB as a powerful approach for dissecting the molecular basis of microbial community growth.

## INTRODUCTION

To colonize environmental and host niches, microbes often form surface-attached multicellular communities called biofilms (1). Biofilms are formed via the secretion of extracellular components that facilitate cell-to-cell and cell-to-surface adhesion (2). The biofilm matrix sterically restricts interactions between the cells and threatening agents such as bacteriophages and antibiotics (3). In addition, resident cells are protected from dislocation by fluid flow, and the high cell density that emerges from cell-to-cell attachments facilitates intercellular interactions such as the exchange of metabolites, cell-cell communication molecules, and genetic material (1). Biofilm-forming bacteria are notorious for causing difficult-to-treat infections, as the biofilm lifestyle allows bacteria to evade the immune system and overcome clinical interventions (4). Thus, over the past several decades, the identification and characterization of molecular mechanisms and ecological principles underlying biofilm formation have become major areas of focus within microbiology.

Given the ubiquity and importance of biofilms in medical and industrial settings, there is a broad interest in assessing the characteristics of biofilms formed by diverse microbial communities, species, and strains in a non-perturbative fashion. Furthermore, in genetically tractable organisms, there is great interest in identifying genes controlling biofilm development, which often requires high-throughput genetic screens. Existing methods for measuring biofilm characteristics are generally invasive, low-throughput, and/or unable to capture temporal dynamics of biofilm development. One of the most widely used methods to assess the propensity of a bacterial culture to form biofilms is the crystal violet (CV) assay (5, 6). In this approach, bacteria are cultured, non-adherent cells are washed away, and the remaining biofilm cells are stained using the CV dye. “Biofilm biomass”—a proxy for the number of biofilm cells in the community—can then be inferred by measuring the absorbance of solubilized CV using a spectrophotometer. While this approach has enabled many discoveries and has been instrumental in progressing the study of biofilms, it imposes significant limitations: (i) the staining process kills cells, making real-time measurements impossible, (ii) the numerous washing steps can dislodge biofilm structures, and (iii) CV staining does not provide any information about biofilm morphology. Another common approach for measuring biofilm properties is confocal, light-sheet, or super-resolution fluorescence microscopy, which, combined with advanced image analysis techniques, can yield reliable quantitative information on both biofilm dynamics and architecture (7–12). However, there are two major caveats to this method: (i) fluorescence-based imaging generally requires genetic manipulation of organisms of interest to introduce fluorescent probes, and (ii) samples are often subject to photodamage and photobleaching during image acquisition. Using confocal fluorescence microscopy in combination with a minimally invasive “fluorescence exclusion” negative-staining approach (13) circumvents these limitations and has recently been applied for high-resolution time-lapse imaging of biofilms (14), but the scalability of this method remains limited. The drawbacks of these and other assays for biofilm development have contributed to a diversity of approaches that are employed in biofilm literature, which, in turn, has led to inconsistent empirical standards for the objects that are qualified as biofilms.

Our previous work explored label-free imaging (e.g., brightfield microscopy) as a simple, generic approach for assessing biofilm dynamics in the gram-negative pathogen *Vibrio cholerae* (*V. cholerae*) (15, 16). Since bacterial cells and biofilm matrix components intrinsically generate optical contrast, label-free microscopy can be broadly used to assess the visible phenotypes of bacterial biofilms across a range of species. It has the advantage of being minimally invasive and does not require genetic manipulation to introduce probes. Moreover, the simple and affordable optics required for brightfield microscopy are readily available to researchers investigating microbial group behaviors across the world. In this work, we demonstrate that low-magnification brightfield imaging of bacterial growth in microtiter plates, together with simple image analysis steps, can be used to reliably quantify microcolony biofilm lifecycles in diverse bacterial species, including gram-positive and gram-negative bacteria. We refer to this approach as “label-free analysis of biofilms” (LFAB). By applying the LFAB approach to multiple species, we show that LFAB measurements of biofilm biomass correlate strongly with ground-truth confocal microscopy and CV staining measurements. Furthermore, we provide a user-friendly image analysis application, which automates the steps required to extract quantitative data from this assay, so that it can be readily adopted by the field.

To demonstrate the power of the LFAB approach, we used high-throughput imaging to discover genes implicated in the biofilm lifecycle of the gram-positive human pathogen *Streptococcus pneumoniae* (*S. pneumoniae*). *S. pneumoniae* is a WHO priority pathogen (17), responsible for an estimated 750,000 annual deaths worldwide (18). It forms biofilms on the host epithelium during chronic colonization of the nasopharynx (19–23) and of the middle ear (24), as well as during chronic heart disease (25, 26). *S. pneumoniae* biofilms are sites for strain evolution by horizontal gene transfer (19, 27) and provide protection from antimicrobials (28, 29) and host immune cells (19, 30, 31). Despite the importance of the biofilm lifecycle for *S. pneumoniae* ecology, few studies have sought to characterize its molecular determinants (32). Moreover, none of these studies has yielded a comprehensive model that describes the developmental stages of *S. pneumoniae* biofilm development.

To address gaps in knowledge of the *S. pneumoniae* biofilm lifestyle, we investigated the dynamics of microcolony biofilm growth, a morphology formed by the radial expansion of founder cells, and identified genes involved in their development. We found that initial cell inoculum, presence/absence of the cell-surface capsule polysaccharide, and nutrient levels strongly determined the propensity of an *S. pneumoniae* population to form microcolony biofilms *in vitro*. Using a transposon (Tn) screen, we further identified over 100 high-value candidate genes implicated in different stages of biofilm development. Among these, a particularly striking phenotype was observed in a strain carrying a Tn-insertion in the gene encoding the cell-surface protein CbpA (33–36), resulting in an almost complete disruption of microcolony biofilms. We therefore investigated the role of CbpA and its regulatory genes (37–39), demonstrating that CbpA promotes late-stage microcolony biofilm stability, and that proper regulation of CbpA is essential for *S. pneumoniae* biofilm developmental dynamics. Another notable phenotype, hyper-biofilm formation, was observed due to Tn-insertion in the gene encoding peptidoglycan hydrolase LytB. Using a clean deletion, we showed that LytB influences the spatial distribution and morphology of *S. pneumoniae* microcolony biofilms. Together, our study highlights the utility of LFAB across multiple bacterial species and validates its ability to identify critical genetic determinants of the biofilm lifestyle using *S. pneumoniae* as a test case.

## RESULTS

### Low-magnification brightfield microscopy of culture growth yields accurate microcolony biofilm quantifications

**Fig 1:**
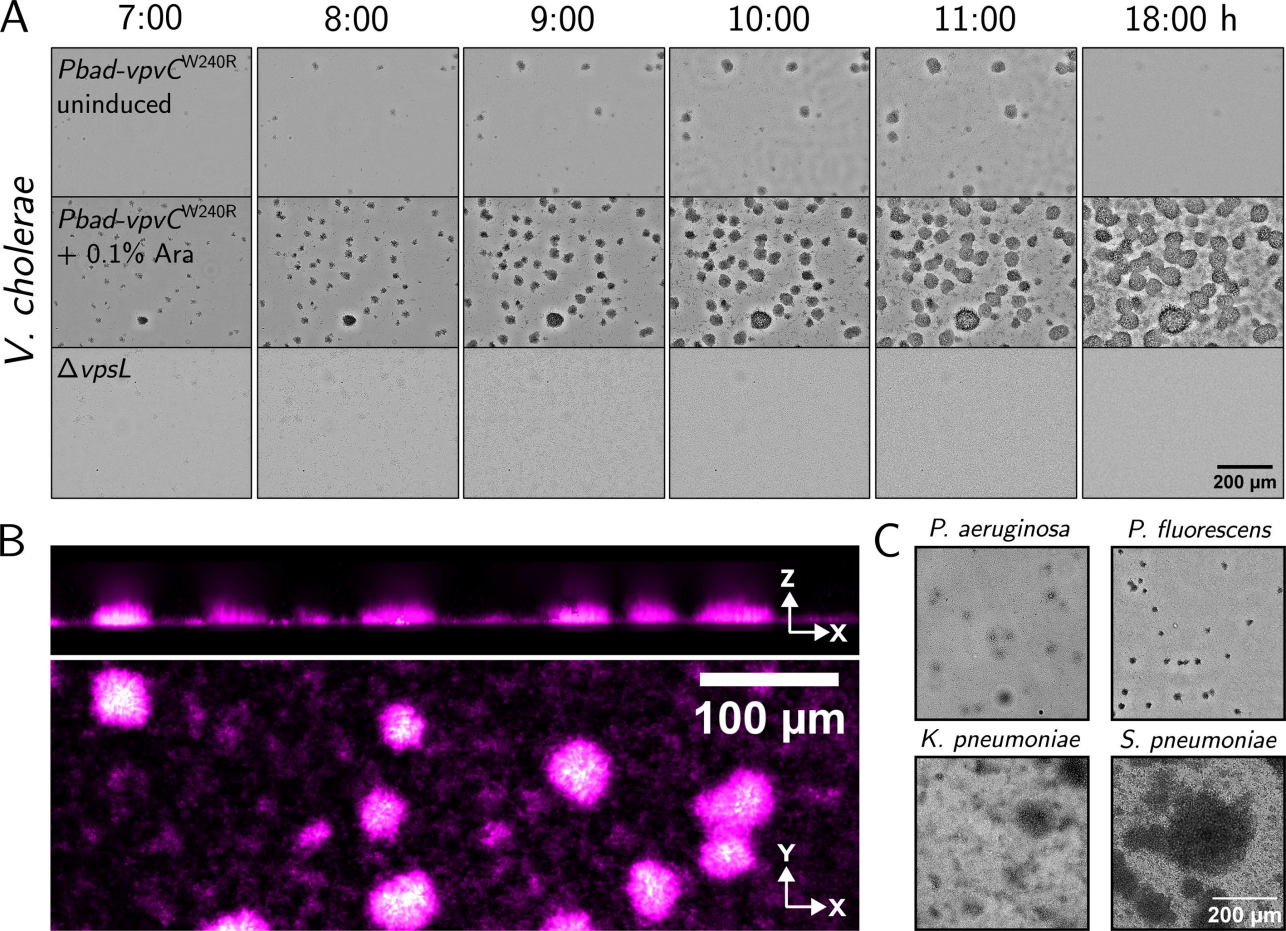
Low-magnification brightfield microscopy captures microcolony biofilm dynamics through regional pixel darkening. (**A**) Time series of brightfield images acquired with a 10x objective for *V. cholerae.* Timepoints reflect the number of hours post-inoculation, and the scale is as indicated. Top panels represent biofilm formation by a strain carrying an arabinose-inducible diguanylate cyclase *(Pbad-vpvC*^W240R^*)* grown in the absence of arabinose (mimicking wild-type conditions). The middle panels represent the same strain induced with 0.1% (wt/vol) arabinose, which drives expression of a constitutively active diguanylate cyclase and thereby activates biofilm formation. Bottom panels represent the Δ*vpsL* strain, which lacks a *Vibrio* exopolysaccharide gene known to be required for biofilm development. Images representative of *N = 5* biological replicates. (**B**) Confocal fluorescence images of the induced *Pbad-vpvC*^W240R^ strain after 24 hours of growth, acquired with a 20× magnification objective. Cells were stained with 10 µM of the lipophilic dye MM4-64. Images are colored in a magenta-hot lookup table. Top: side-on *x-z*-projection. Bottom: single *x-y* plane. Image is representative of *N = 3* biological replicates. Scales and axes are as indicated. (**C**) Brightfield images of the indicated species (strains: *P. aeruginosa* PAO1Δ*wspF*, *P. fluorescens* SBW25Δ*wspF*, *K. pneumoniae* KPPR1Δ*wcaJ*, *S. pneumoniae* SV36 unencapsulated), acquired with a 10× magnification objective at the solid-liquid interface after 24 hours of culture growth. The scale bar is the same for all species in panel **C** and is shown in the bottom right panel. Images are representative of *N* = 3 biological replicates with *n* = 3 technical replicates each.

We initiated LFAB development by imaging microcolony biofilms formed by the pathogen *V. cholerae* using low-magnification brightfield microscopy without perturbation (i.e., no washing). To investigate biofilm dynamics, we used a *V. cholerae* strain carrying *Pbad-vpvC*^W240R^, which encodes an arabinose-inducible diguanylate cyclase. Consistent with previous reports (8–10), when we inoculated dilute cultures of this strain under uninduced conditions (mimicking a wild-type (WT) *V. cholerae*), we observed microcolony biofilm formation at the solid-liquid interface (i.e., at the bottom of microtiter dishes), which peaked at ~11 hours post-inoculation, followed by biofilm dispersal (Fig. 1A; Movie S1). When the same strain was induced with 0.1% arabinose, we observed robust microcolony biofilm formation at the solid-liquid interface and no dispersal (Fig. 1A; Movie S2). In brightfield images, microcolony biofilms appeared as darkened regions due to increased light scattering compared to background regions. We confirmed the verticalization of these biofilm communities using confocal microscopy (Fig. 1B). To verify that regional darkening in brightfield images is dependent on verticalized biofilm growth, we captured time-lapses of a mutant lacking an exopolysaccharide biosynthesis gene (*vpsL*) known to be required for biofilm formation (40). As expected, these cultures exhibited homogeneous growth across the field of view with no regional heterogeneity in transmittance (Fig. 1A). Notably, we found that other biofilm-forming bacteria, including *S. pneumoniae*, *Klebsiella pneumoniae*, and *Pseudomonas* species, also exhibited microcolony biofilm formation, with distinct morphologies, at the solid-liquid interface when grown under similar conditions (Fig. 1C).

**Fig 2:**
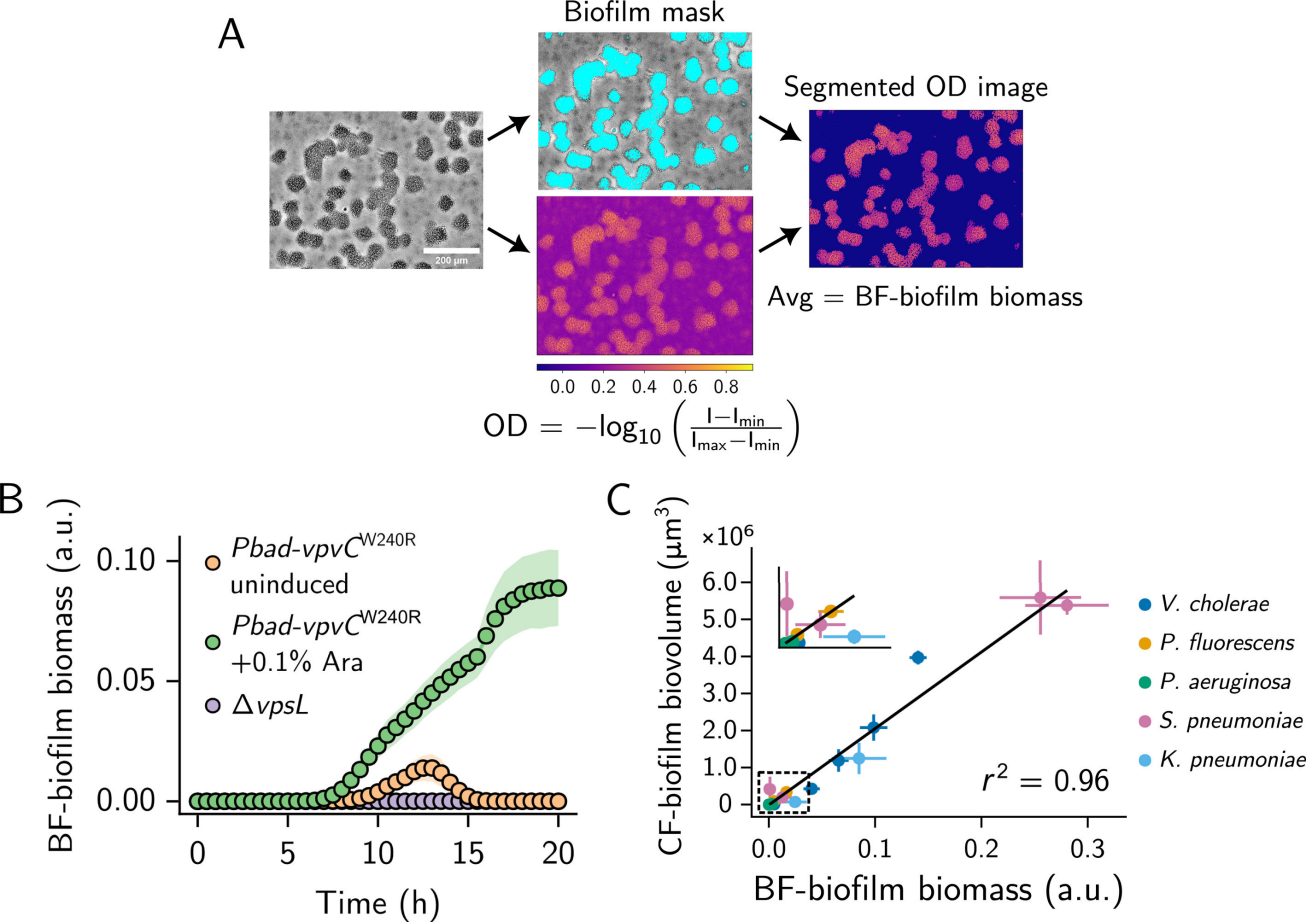
Proof-of-principle for using low-magnification brightfield microscopy of culture growth as a species-agnostic and quantitative biofilm assay. (**A**) Schematic of the pipeline for analyzing brightfield images of culture growth in microtiter plates. Images are from a *V. cholerae* strain carrying a chromosomal *Pbad-vpvC*^W240R^ construct, induced with 0.1% (wt/vol) arabinose (41). Cultures were grown for 24 hours from an initial density of ~10^4^ cells/mL. Left: raw image. Middle, top: raw image with a binary mask showing biofilm regions overlaid in cyan. The mask was computed as indicated in Results and Materials and Methods. Middle, bottom: optical density (OD) image calculated based on the indicated transformation, applied pixel-wise to the raw image. Right: the segmented OD image is generated by applying the mask to the OD image. The average over all pixels of this image gives us the BF-biofilm biomass. The following represent pixel intensities of respective images: “*I*” = raw image; “*I*_min_” = image acquired with the camera shutter closed; “*I*_max_” = image of fresh media (i.e., a blank). (**B**) Time series of biofilm biomass values, calculated from images of the indicated strains over a 20-hour lifecycle. The *Pbad-vpvC*^W240R^ strain carries an arabinose-induced diguanylate cyclase that drives biofilm formation. The uninduced condition is in the absence of arabinose (mimicking wild-type conditions), whereas induction with 0.1% (wt/vol) arabinose drives the expression of a constitutively active diguanylate cyclase and thereby activates biofilm formation. Lines represent means, and shading represents standard deviation. *N* = 5 biological replicates. (**C**) Correlation of biofilm biomasses, calculated by brightfield microscopy using a 4× magnification objective (*x*-axis) and biofilm biovolume, calculated by spinning-disc confocal fluorescence microscopy using a 20× magnification objective (*y*-axis), for the indicated species (strains: *P. aeruginosa* PAO1 WT and Δ*wspF*; *P. fluorescens* SBW25 WT, Δ*wspF*, and Δ*wssA-J*; *K. pneumoniae* KPPR1 WT and Δ*wcaJ*; *S. pneumoniae* D39 and SV36 encapsulated and unencapsulated; *V. cholerae* Δ*vpsL* and *Pbad-vpvC*^W240R^ with arabinose concentrations 0%, 0.025%, 0.0375%, 0.05%, and 0.1%). For confocal microscopy, cells were stained with 10 µM (or 20 µM for *S. pneumoniae*) of the lipophilic dye MM4-64. Each point represents the mean biofilm biomass of a strain/arabinose condition, and error bars represent standard deviation. For all strains, parallel cultures were imaged by both confocal fluorescence microscopy and brightfield microscopy. The line represents the best-fit orthogonal distance regression to the data; *r*^2^ = 0.96 based on ordinary least squares. Inset displays the data points in the boxed region, with re-scaled axes. For brightfield microscopy in panel C, *N* = 3 biological replicates with *n* = 3 technical replicates each. For confocal microscopy, *N* = 3 biological replicates. a.u.: arbitrary units. OD: optical density. BF: brightfield. CF: confocal fluorescence. Ara: arabinose. Scale bar as shown.

Our next goal was to exploit the transmittance information present in our low-magnification brightfield images to quantify microcolony biofilm biomass and to compare these measurements to ground-truth biofilm biovolume measured by confocal fluorescence microscopy. We set up an image analysis pipeline toward this goal. As noted above, microcolony biofilms appear as regions of low intensity on brightfield images since they scatter more light than their surrounding areas, which consist of comparatively sparse planktonic or non-biofilm-associated cells. As a result, we found that microcolony biofilms in our images could be reliably thresholded and their transmittance properties could be measured using simple image analysis techniques (depicted schematically in Fig. 2A; for details, see Materials and Methods). First, to identify biofilm regions, a local background subtraction was applied to raw images, blurring was performed to eliminate intrinsic intensity variation inside biofilms, and a constant intensity threshold was applied to isolate biofilm regions (depicted as a binary mask on the image). Separately, to convert pixel-wise intensity values in the raw images to optical density (OD) values, a log_10_-based transformation was applied to the transmittance, which accounted for the maximum transmittance (a media-only blank) and the camera noise of the optical configuration. Finally, the binary biofilm mask was applied to the optical density image, yielding a segmented biofilm image, where pixel intensities represent the degree of light scattering within biofilm structures. As a measure of the overall biofilm biomass across the field of view (accounting for biofilm surface area coverage and biofilm transmittance), the average optical density of the segmented biofilm image was computed, which we hereafter refer to as brightfield (BF)-biofilm biomass. We provide a free, user-friendly web application (see https://github.com/BridgesLabCMU/Brightfield-biofilm-assay), so that our brightfield image analysis pipeline is accessible to all biofilm researchers.

LFAB provides a relative optical readout rather than a strictly quantitative physical measurement, since the output is derived from intensity values and therefore depends on all factors that affect brightfield optics (e.g., growth medium, microscope, objective lens, and acquisition settings). As a result, quantitative comparisons are only appropriate when measurements are performed under identical experimental conditions and should be interpreted relative to an internal control, as in CV staining. Additionally, BF-biofilm biomass is derived from single-plane imaging, but since light is captured from a depth of field extending beyond just the focal plane, the calculated biomass reflects a combination of underlying three-dimensional attributes, including biofilm cell density and biovolume. The quantitative mapping between BF-biofilm biomass and these attributes is not known *a priori* and will again depend on optical and experimental parameters. Accordingly, throughout this work, we compare BF-biofilm biomass values only among samples imaged under identical conditions and do not assign physical meaning to individual BF-biofilm biomass values in isolation.

A major advantage of our approach is that it is non-perturbative (washing is not necessary), and measurements can be made in real time at high temporal resolution throughout biofilm growth. To demonstrate this utility, we captured brightfield images during culture growth and measured biofilm biomass at each timepoint (Fig. 2B). Under biofilm-inducing conditions in *V. cholerae,* that is*,* overexpression of the diguanylate cyclase, we observed that microcolony biofilm biomass increased post-inoculation steadily, eventually plateauing at ~18 hours (presumably when nutrients become limited). The same strain, when uninduced (mimicking wild-type), exhibited biofilm growth peaking at 11–12 hours post-inoculation, followed by rapid dispersal, as shown previously (Fig. 2B) (8–10). By contrast, the exopolysaccharide-deficient strain exhibited no appreciable microcolony biofilm biomass over the entire growth cycle (Fig. 2B). These results suggested that brightfield microscopy of culture growth in microtiter plates, combined with simple image analysis steps, can be used to quantify entire biofilm development cycles in real time.

Having established a pipeline to measure biofilm biomass from brightfield images, we validated our approach by comparing the brightfield quantifications to biofilm biovolume measurements from confocal fluorescence microscopy. Additionally, we wanted to assess the utility of this pipeline for other biofilm-forming bacteria. To this end, we analyzed brightfield images for various biofilm formers under a range of biofilm-inducing conditions (*V. cholerae*, *P. fluorescens*, *P. aeruginosa*, *K. pneumoniae*, and *S. pneumoniae*). Concurrently, we measured the total cellular biofilm biovolume for the same strains/conditions using volumetric spinning-disc confocal fluorescence microscopy, in which biofilm cells were labeled with the lipophilic dye, MM4-64, as a ground truth for comparison. We found a strong correlation (*r*^2^ = 0.96 based on ordinary least squares) between brightfield biofilm biomass and confocal biofilm biovolume across all strains and species (Fig. 2C). Qualitatively, the correlation between brightfield biomass and confocal biovolume was independent of the objective lens (as long as sufficiently low-magnification objective lenses were used) (Fig. S1A) or the microscope (Fig. S1B), thus demonstrating that the analyses are agnostic to microscope hardware. It must be noted, however, that the correlation between brightfield imaging and confocal biovolume measurements may become weaker if more of the biofilm lies outside of the depth of field from which the objective lens collects light during brightfield imaging. Therefore, we recommend estimating the depth of field of the objective lens being used and comparing it to approximate heights of the biofilms to make more informed interpretations of the quantitative output of LFAB. In addition to validation with confocal biofilm biovolume, we found a strong correlation between brightfield biofilm biomass measurements and absorbance due to CV staining (Fig. S1C; *r*^2^ = 0.84 based on ordinary least squares). Together, our results show that analyses of biofilm images captured by brightfield microscopy can recapitulate the results of confocal fluorescence microscopy or CV staining and can enable tracking of biofilm development at high temporal resolution.

During image acquisition, a relevant control parameter is the focal position. We found that when imaging small biofilms, biofilm biomass values were affected considerably by focal position, whereas this effect was attenuated when imaging larger biofilms (Fig. S2A). Thus, establishing a single focal position is necessary to ensure consistency across experiments that would enable quantitative comparisons. Moreover, we found that in cases where populations were comprised of a large fraction of planktonic cells, planktonic cell light scattering led to lower biofilm biomass values due to their homogenizing effect on local image intensities, which impedes the detection of microcolony biofilms (Fig. S2B). Thus, we caution against an interpretation of the absolute fraction of dispersed cells in cases where populations undergo biofilm maturation-dispersal lifecycles. In cases where it is desirable to compute such fractions, we recommend endpoint washing. Planktonic cell effects, together with focal position effects, comprise the major sources of non-biological quantitative variance within the LFAB framework.

### Characterization of *S. pneumoniae* microcolony biofilms using LFAB

**Fig 3:**
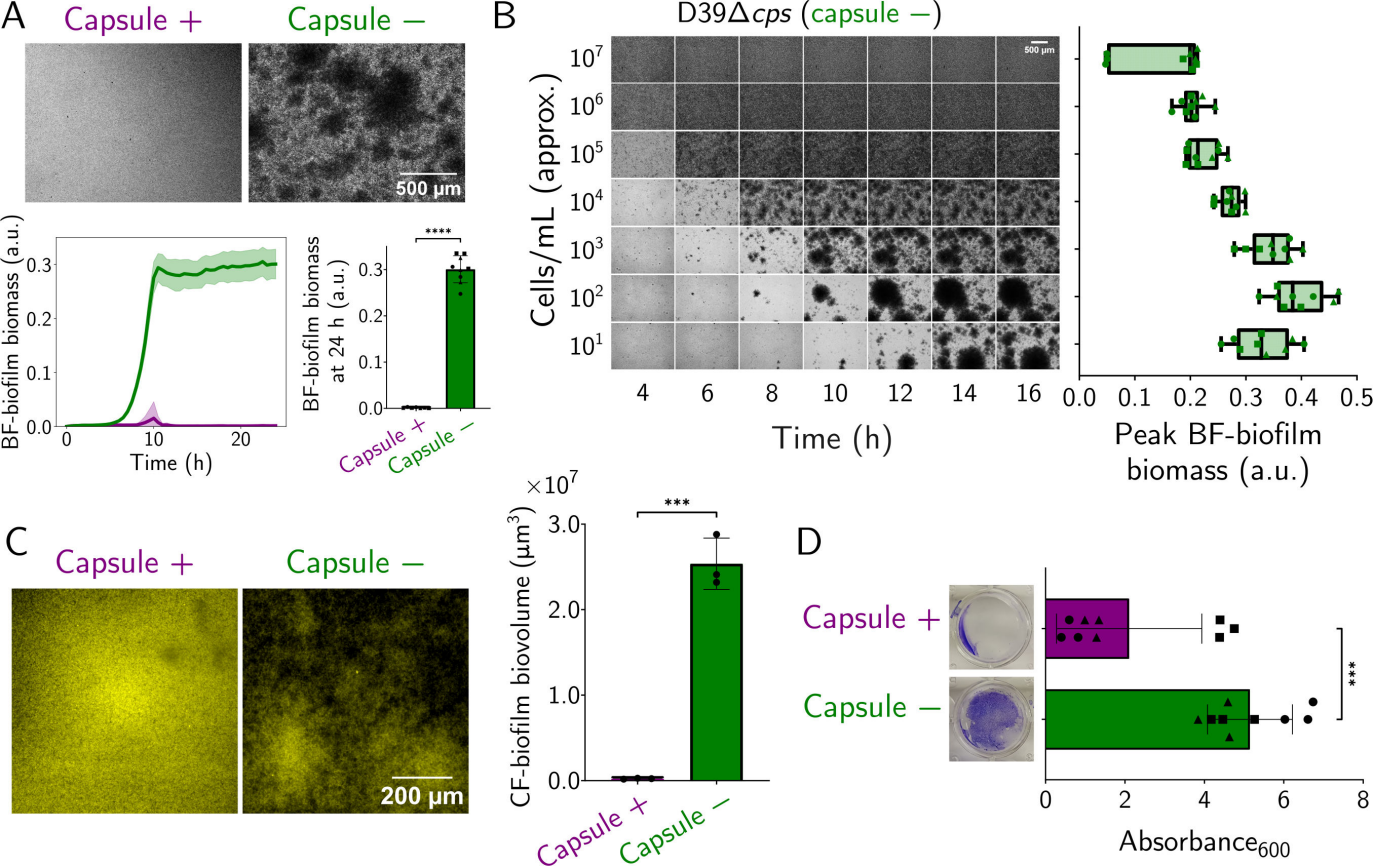
Absence of capsule and low seeding density enable microcolony biofilm formation in *S. pneumoniae* (model strain D39). (**A**) Brightfield microscopy images (4× magnification) of model strain D39 WT (type 2 capsule; left) and isogenic unencapsulated (D39Δ*cps*) mutant (right) at 24 hours post-seeding, grown from ~10^3^ cells/mL. Time-series plot (bottom) shows microcolony biofilm biomass for WT (purple, capsule +) and Δ*cps* (green, capsule –) strains, quantified by LFAB from 0 to 24 hours post-seeding at 30-min intervals. The line shows the mean, and the shaded region shows the standard deviation. Bar plot (bottom right) shows BF-biofilm biomass at the 24-hour timepoint. (**B**) Time-series brightfield images (left) of D39Δ*cps* at various initial cell seeding densities, showing the highest seeding density at the top. Points on the boxplot (right) show the peak microcolony biofilm biomass for respective seeding densities across a 24-hour time series. Box plots show median and interquartile range, and whiskers show min and max values. (**C**) Confocal microscopy images (showing *Z*-stack sum projection) of D39 WT (left) and Δ*cps* (right) strains. Biofilms were stained with 20 µM of the lipophilic dye MM4-64 and imaged at 24 hours post-seeding at 20× magnification. Bar plot shows quantified cellular biovolume of microcolony biofilms. (**D**) CV assay for D39 WT and Δ*cps* at 24 hours post-seeding. Biofilms were washed three times with phosphate-buffered saline (PBS), stained with 0.1% CV, and excess stain was removed with three more PBS washes. Images show representative wells after staining. CV was then quantified by solubilizing in 70% ethanol and measuring absorbance at 600 nm on the spectrophotometer. For LFAB and CV assays, *N* = 3 biological replicates with *n* = 3 technical replicates each; each point on the bar plot is a technical replicate, and each biological replicate is shown by a unique symbol. For confocal microscopy, *N* = 3 biological replicates; each point on the bar plot is a biological replicate. Scale bars are as indicated. (**C and D**) Bars show the mean, and error bars show the standard deviation. Student’s two-tailed *t*-test; ***P < 0.001; ****P < 0.0001. BF: brightfield. a.u.: arbitrary units.

To demonstrate the utility of LFAB, we chose to further interrogate the biofilm lifecycle of *S. pneumoniae*. *S. pneumoniae* forms biofilms during asymptomatic chronic colonization of the upper respiratory tract (19–23), and during invasive disease in the middle ear (24) and the heart (25, 26). Our goal was to resolve the phases of *S. pneumoniae* biofilm development and to identify molecular determinants controlling the lifecycle. *S. pneumoniae* expresses a polysaccharide capsule on the cell surface, which is a major virulence determinant. Multiple studies have reported an inhibitory role for the capsule in *in vitro* biofilm development (19, 27, 29, 42–44). *In vivo*, the capsule is downregulated when cells adhere to host epithelial cells, such that low capsule conditions are relevant to colonization (19, 45). Thus, we began by searching for an optimal seeding density in unencapsulated variants of two *S. pneumoniae* strains (model strain D39 and clinical strain SV36). Our aim was to identify conditions that allowed us to assess the ability of individual founder cells to grow into verticalized microcolony biofilms. We captured brightfield time-lapses and observed that when seeding these unencapsulated strains at low density (10^4^–10^1^ cells/mL), distinct cell clusters were observable 5–6 hours post-inoculation and grew radially into microcolony biofilms that plateaued at a peak biomass around 12 hours after inoculation (Fig. 3A and B; Fig. S4A and B; Movies S3 and S4).

We investigated the role of nutrient levels, seeding density, and capsule in microcolony biofilm formation. We found that diluting the growth medium decreased the plateau biomass, suggesting that the plateau is, in part, determined by nutrient limitation (Fig. S5). Moreover, growth in nutrient-rich medium produced less defined microcolony biofilms with a lower peak biomass (Fig. S5), consistent with previous findings that *S. pneumoniae* biofilm formation is inhibited in high-nutrient conditions (19, 27, 42, 46). We then systematically tested the effect of seeding density on microcolony biofilm formation. We found that in contrast to the microcolony biofilms observed at low seeding density, when seeded at high densities (10^5^ cells/mL and above), unencapsulated strains grew into a confluent structure covering the entirety of the field of view (Fig. 3B; Fig. S4B). Finally, while unencapsulated strains displayed the ability to form microcolony biofilms at low seeding density, encapsulated strains did not grow into microcolony biofilms at any density, suggesting that their propensity to form verticalized biofilms from single founder cells is reduced (Fig. 3A; Fig. S3; Fig. S4A). These results were confirmed by confocal fluorescence microscopy, demonstrating that differences in microcolony biofilm formation between encapsulated and unencapsulated strains cannot be attributed to differences in brightfield optical properties of the capsule (Fig. 3C; Fig. S4C). Previous work suggests that encapsulated strains form confluent adherent biofilms at high densities (29, 43, 44, 47–49), but we are unable to assess the confluent morphology using the LFAB imaging approach due to the lower optical contrast generated by these structures. Thus, we conclude that microcolony biofilms are sensitive to the concentration of nutrients, and are best observed using strains where the capsule is downregulated and seeding density is low.

There is extensive genomic diversity across *S. pneumoniae* strains. Indeed, D39 and SV36 are highly divergent, yet unencapsulated SV36 formed robust microcolony biofilms with high biomass, and few bacteria outside of the biofilms (compare Fig. 3A and Fig. S4A). To extend this to another strain, we investigated whether the microcolony biofilm phenotype is conserved in a second model strain (TIGR4). In contrast to D39 and SV36, under identical conditions, the model strain TIGR4 displayed the confluent phenotype, even when using an unencapsulated strain at low seeding density (Fig. S6A). However, when grown in diluted growth media, the unencapsulated TIGR4 formed microcolony biofilms, although to a much lesser extent than the unencapsulated D39 or SV36 strains (Fig. S6B). Together, these findings suggest that the microcolony biofilm phenotype is exhibited by multiple strains under different nutrient conditions and exhibits strain-specific variability.

We note that the microcolony biofilms examined here for all *S. pneumoniae* strains are grown under conditions that are quite distinct from the conventional conditions in the field. *S. pneumoniae* biofilms have traditionally been examined after inoculation at comparatively high densities using a diverse set of strains, many expressing capsular genes, generally yielding confluent biofilms (29, 43, 44, 47–50). These biofilms are often quantified using CV assays (32, 48) or by confocal microscopy (43, 51). Thus, the *S. pneumoniae* microcolony biofilms analyzed here represent a mode of biofilm growth that, to our knowledge, has not been systematically explored in this organism. To compare our findings with LFAB to established methods, we performed confocal microscopy and CV assays at low seeding densities with the encapsulated D39 and SV36 strains and their respective unencapsulated mutants. The CV assays and confocal microscopy mirrored the phenotypes observed by LFAB, both for D39 (Fig. 3C and D) and for SV36 (Fig. S4C and D). We conclude that the *S. pneumoniae* microcolony biofilms captured by LFAB faithfully represent biofilm-forming capacity as defined by established assays while also unlocking new opportunities to study biofilm dynamics in real time and at scale.

### High-throughput screening for molecular determinants of *S. pneumoniae* microcolony biofilms

A major advantage of the LFAB methodology is that it can easily be scaled to image and analyze hundreds to thousands of samples in a day using robotic microscopy. Having established a robust protocol yielding microcolony biofilms that can be reliably quantified using LFAB, we turned towards utilizing the platform’s high-throughput capability to identify genetic determinants of microcolony biofilm formation in *S. pneumoniae*. A previous study reported genes associated with biofilm formation in *S. pneumoniae* using a CV-based screen of a transposon library (32). Here, we revisited this approach using LFAB, with the added advantage of being able to utilize temporal information of biofilm dynamics in the screening process. To this end, we prepared an indexed library of transposon (Tn) insertion mutants in the unencapsulated model strain D39Δ*cps*. We selected this background for two reasons. First, D39 displays high transformation efficiency (>10-fold higher than most clinical strains, including SV36). Second, as a widely used model strain, there is extensive literature in this strain background, allowing for curation of our hits with published data (52–54).

**Fig 4:**
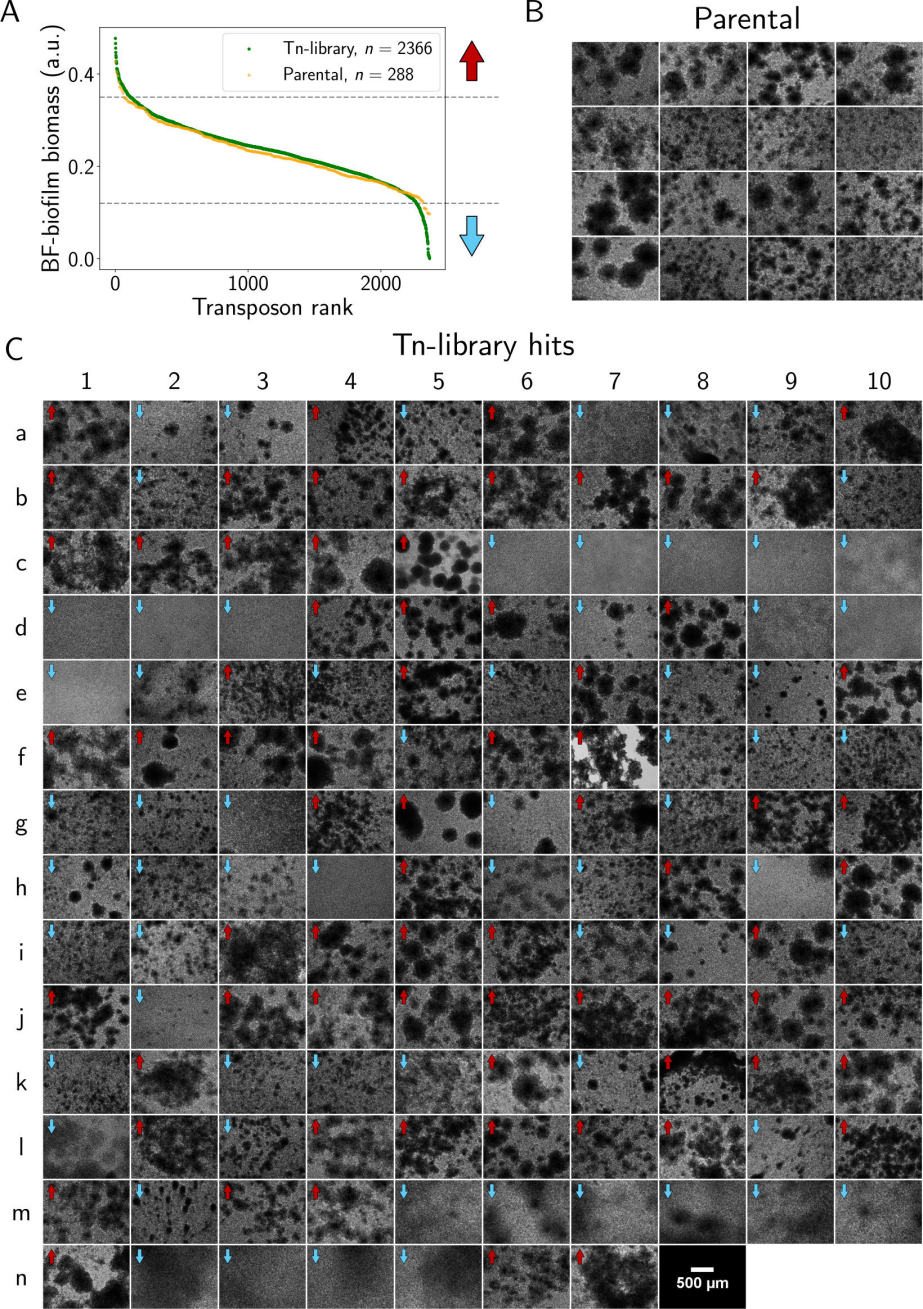
A brightfield microscopy screen identifies novel genes implicated in biofilm development in *S. pneumoniae*. (**A**) Rank-ordered distribution of all Tn-mutant clones screened for microcolony biofilm phenotype at 24 hours post-seeding (green points). The *y*-axis displays the BF-biofilm biomass quantified by LFAB, and the *x*-axis shows the rank of each Tn-mutant clone when arranged in decreasing order of biofilm biomass. Yellow points display replicates of the parental strain (D39Δ*cps*) imaged in an identical fashion to the Tn-library, forming a “null” distribution. Gray dotted lines show the microcolony biofilm biomass thresholds (0.35 and 0.12), on either side of which we shortlisted Tn-mutants for performing time-lapse imaging. (**B**) Representative images (at 24 hours) of parental strain (D39Δ*cps*), capturing variability in inoculum due to high-throughput processing by a liquid-handling robot. (**C**) Endpoint (24 hours) images of all 137 Tn-mutant clones that were significantly different from the parental (66 below and 71 above the parental biofilm biomass threshold at endpoint) from the Tn-library and were thus considered hits of the screen. Details of clones are given in Table S1. Corresponding biomass time-series trajectories are shown in Fig. S7. The blue downward arrow indicates lower microcolony biomass than parental, and the red upward arrow indicates higher microcolony biofilm biomass than parental, as determined by endpoint imaging. Position n8 is blank and shows the scale bar for all images in panels** B** and **C**. BF: brightfield. Tn: transposon.

To screen for biofilm phenotypes, we seeded low-density cultures (diluted to ~10^3^ cells/mL using a liquid-handling robot) from each of the 2366 Tn-mutant clones in our library into 96-well microtiter plates. These cultures were grown for 24 hours, after which they were imaged, and biomass was quantified using LFAB. To set the baseline for D39Δ*cps* (parental strain of the library) and account for inherent variability between replicates, we imaged and quantified 288 replicates of the parental strain in the same manner (representative images in Fig. 4B). We then plotted the distributions of biofilm biomass for the parental strain (Fig. 4A, yellow) and the Tn-mutant clones (Fig. 4A, green) in rank order. Using low-stringency thresholds on this distribution (all mutant clones beyond the ~2.5% tails on either end of the parental distribution), we shortlisted 213 mutant clones that displayed higher or lower microcolony biofilm biomass than the parental strain (see dotted lines in Fig. 4A). To incorporate temporal information into the screen, we next performed time-lapse imaging of these 213 shortlisted mutants in biological duplicate and used LFAB to quantify the dynamics of biofilm development. As a baseline for comparison, we imaged and quantified time-lapses for 180 replicates of the parental D39Δ*cps* strain and calculated the mean and standard deviation (SD) of the resulting BF-biofilm biomass curve. To identify Tn-mutants that significantly differed from the parental strain, we compared each mutant’s biomass trajectory against this baseline. Any mutant whose average BF-biofilm biomass trajectory (from two replicates) fell outside of the parental mean ± SD at any timepoint (i.e., |*Z*-score| > 1 at any timepoint) was considered significantly different from the parental strain (Fig. 4C and Fig. S7, for endpoint images and biofilm biomass trajectories, respectively). This analysis yielded 137 hits (5.8% of the total library), of which 66 displayed reduced and 71 displayed increased biofilm biomass relative to the parental strain at endpoint. Using Sanger sequencing, we identified the transposon insertion locations in each of these mutants and found that they mapped to 120 unique open reading frames (Table S1). Visual inspection of the images revealed that the Tn-mutants display several distinct microcolony morphologies, with differences in features such as microcolony biofilm size, shape, edge structure, spacing between microcolonies, and distribution of interstitial non-microcolony cells (Fig. 4C), which will be quantified in future work. The genes identified in our screen as modulators of *S. pneumoniae* biofilm formation included components with established roles in regulating *S. pneumoniae* biofilms (which validated our approach), as well as genes that have never been characterized (Table S1).

We identified multiple hits in carbohydrate transporters and modifiers. This class of genes is central to *S. pneumoniae*, which relies exclusively on carbohydrates as a carbon source, a feature reflected in the high number of sugar transporters encoded in its genome (55, 56). In addition to its critical role as a nutrient, some carbohydrates are also structural components of the capsule (57) or of the cell wall (58). Genes in this functional category have been highlighted in previous work to affect biofilms based on CV staining (32), but our screen revealed multiple new components. For example, we found that an insertion in the galactose catabolism gene (*galT*) (Fig. 4C, position j8) or the UDP-galactose epimerase *galE* (Fig. 4C, position j7) led to an enhancement in biofilm production. We also found that an insertion in the glycogen synthase *glgA* reduced biofilms (Fig. 4C, position g2). Most striking was the observation that seven clones with insertions in a mannitol importer locus displayed a dramatic drop in biofilm biomass by 24 hours (Fig. 4C, positions c7–c10, d1–d3). Overall, the number of hits in carbohydrate-related genes suggests that *S. pneumoniae* links metabolic information to its biofilm lifestyle.

We also observed a dramatic loss of biofilms at 24 hours due to insertion within the cell wall biosynthetic enzyme gene *murM* and its operon (Fig. 4C, positions d10, e1). This tRNA-dependent aminoacyl transferase plays a critical role in the assembly of branched peptidoglycan on the cell wall (59, 60). MurM is required for resistance to penicillin (60–62), highlighting its importance on the cell surface. More recently, MurM has also been shown to buffer entry into the stringent response (63). The genes encoding *murM*, as well as the peptidoglycan biosynthesis enzymes *murB* and *murE*, have been previously observed in a CV-based screen for biofilm determinants (32). Interestingly, we also found that insertion within the gene encoding another cell wall biosynthetic enzyme, the transglycosylase *pbp2a*, resulted in increased biofilm biomass (Fig. 4C, position k9). Together, these findings suggest that genes coordinating cell wall synthesis are linked to biofilm development. Similarly, our screen and a previous screen (32) both identified cardiolipin synthase, suggesting the composition of the cell membrane may also contribute to biofilm development. Taken together, applying LFAB screening to *S. pneumoniae* revealed a systems-level connection between fundamental *S. pneumoniae* processes and its biofilm lifecycle.

### CbpA and its regulators are critical for microcolony biofilm development

**Fig 5:**
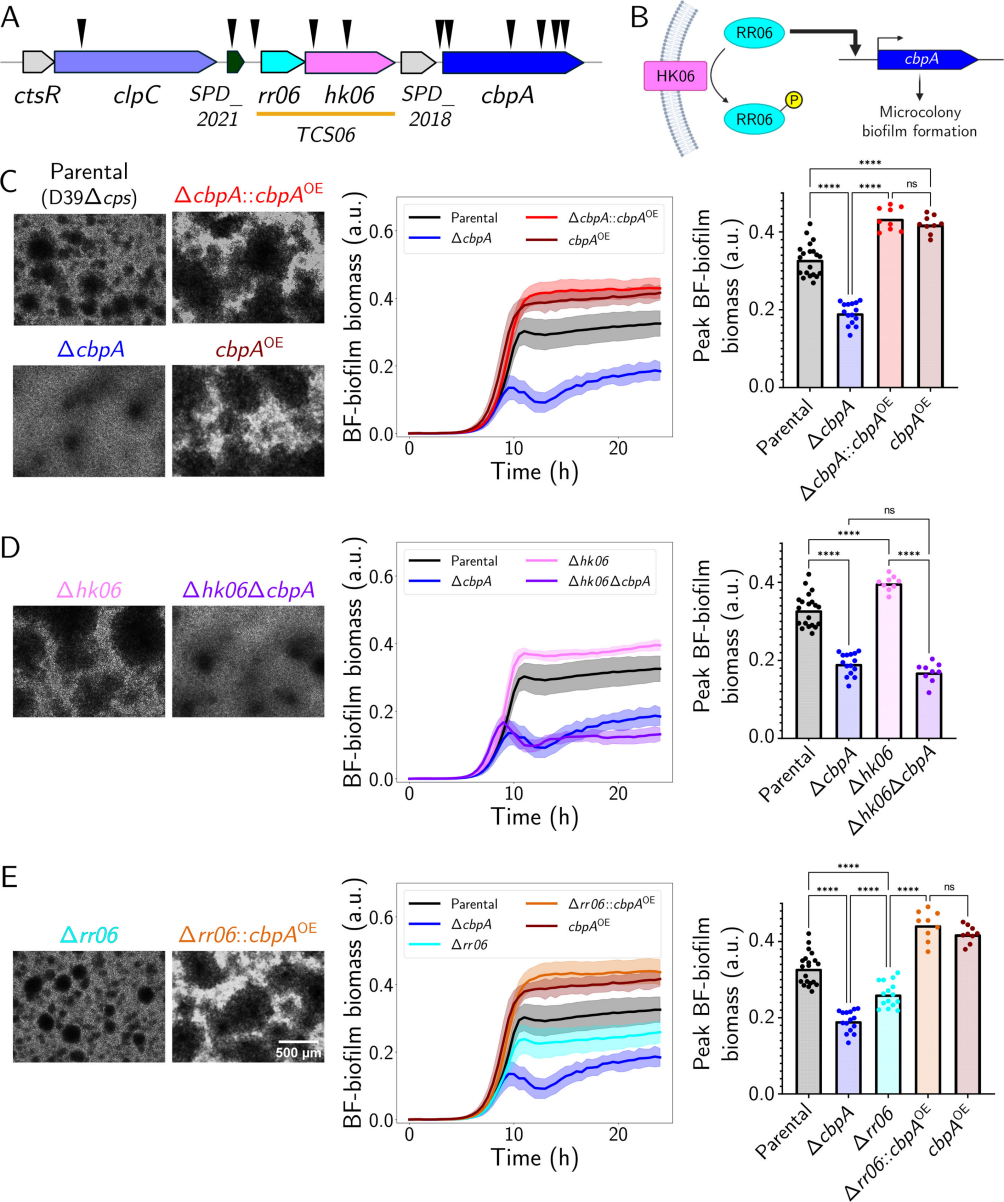
Cell surface protein CbpA and its transcriptional regulation are important determinants of microcolony biofilms in *S. pneumoniae*. (**A**) Schematic of the locus encoding *cbpA*, *TCS06*, and *clpC* in *S. pneumoniae* model strain D39Δ*cps*. Black arrowheads show locations of hits from Tn-screen. *SPD_2018* and *cbpA* form an operon (38), and *rr06* and *hk06* form a separate operon (37), both separate from *ctsR* and *clpC* (64). The operon status of *SPD_2021* is unknown. (**B**) Schematic showing regulation of *cbpA* expression by the two-component system TCS06. The response regulator (RR06) in unphosphorylated form binds to the *cbpA* promoter region and activates its expression, which, in turn, promotes microcolony biofilm formation. Once phosphorylated by the histidine kinase (HK06), phosphorylated RR06 can no longer activate *cbpA* expression (37–39). (**C**) Left: brightfield biofilm images (4× magnification) of the parental strain (D39Δ*cps*), Δ*cbpA*, an overexpressor (OE) complement Δ*cbpA*::*cbpA*^OE^, and a parental *cbpA* OE, at 24 hours post-seeding. Corresponding time-lapse images are shown in Fig. S9. Middle: time series of microcolony biofilm biomass for the above strains, as quantified by LFAB from 0 to 24 hours post-seeding at 30-min intervals. Right: peak microcolony biofilm biomass for each strain. (**D**) As in panel **C**, but for Δ*hk06* and Δ*hk06*Δ*cbpA* strains. Data of parental and Δ*cbpA* controls are replotted from panel **C** for comparison. (**E**) As in panel **C**, but for a Δ*rr06* strain and a *cbpA* OE strain in a Δ*rr06* background. Data of parental, Δ*cbpA*, and *cbpA*^OE^ controls are replotted from panel **C** for comparison. For all strains, *N* = 3–10 biological replicates, with *n* = 3 technical replicates each. Scale bars are the same for all images, as shown in panel **E**. In time-series plots, lines show the mean of all biological and technical replicates, and shaded areas show the standard deviation. In bar plots, each data point represents the peak biomass of an individual time series. One-way ANOVA, *P* < 0.0001 for each set. Šídák’s multiple comparisons test; ****P < 0.0001; ns: not significant. BF: brightfield. a.u.: arbitrary units.

Our next goal was to expand our understanding of *S. pneumoniae* biofilm development by engaging in a detailed genetic characterization of a hit from the LFAB screen, using clean deletion mutants and overexpression mutants. Our screen identified six mutants with Tn-insertions inside/near the gene encoding choline-binding protein A (*cbpA*), all of which displayed an almost complete loss of microcolony biofilms at 24 hours post-inoculation (Fig. 4C, positions m5–m10). In addition, we repeatedly hit genes encoded adjacent to *cbpA*, including a two-component system (*TCS06* or *cbpRS*) and a putative peptide, *SPD_2021* (see locus schematic in Fig. 5A). The dramatic phenotype of the *cbpA* Tn-mutant, combined with hits in adjacent genes with regulatory roles, provided an opportunity to explore the role of a major surface protein and its regulation in *S. pneumoniae* biofilm development.

CbpA is a virulence determinant as well as an abundant surface protein that serves as an adhesin to host cells (33–36). Moreover, two previous studies have reported that a Tn-insertion mutation within the *cbpA* gene of unencapsulated *S. pneumoniae* leads to decreased CV staining at biofilm endpoints (32, 42). To definitively test and characterize the role of *cbpA* across biofilm development, we generated a deletion mutant (Δ*cbpA*) and an overexpression strain (*cbpA*^OE^) and used time-lapse imaging followed by LFAB quantification to establish how changes in *cbpA* expression levels influence microcolony biofilm morphology and developmental dynamics. Consistent with our Tn-insertion mutants, endpoint images from the time-lapses showed that the Δ*cbpA* strain was severely impaired in microcolony biofilm formation (Fig. 5C). Analyses of microcolony biofilm growth and maturation over time revealed that until ~8–9 hours post-inoculation, the Δ*cbpA* and parental strain displayed similar biofilm growth dynamics (Fig. S8). After this point, the Δ*cbpA* strain deviated from the parental phenotype, resulting in a significantly reduced biofilm biomass at later timepoints, as well as an altered, “looser” biofilm morphology (Fig. 5C; Fig. S8; Movies S3 and S5). This phenotype could be complemented by overexpressing *cbpA* from an ectopic genomic location. In fact, the Δ*cbpA*::*cbpA*^OE^ strain displayed greater microcolony biofilm biomass than the parental strain and a decrease in interstitial non-microcolony cells, as noted by reduced light scattering in background regions of images (Fig. 5C; Fig. S9). The parental strain overexpressing *cbpA* (*cbpA*^OE^) mimicked the phenotype of the Δ*cbpA*::*cbpA*^OE^ overexpressor (Fig. 5C; Fig. S9). Together, these data demonstrate that *cbpA* is a molecular determinant of microcolony biofilm formation in *S. pneumoniae* that appears to play a role at later timepoints in stabilizing microcolony biofilm structures, likely by facilitating cell-to-cell adhesion.

Next, we turned our focus to the two-component system *TCS06*, consisting of the histidine kinase *hk06* and the response regulator *rr06*. *TCS06* is encoded adjacent to *cbpA* and is known to regulate its transcription by a mechanism established in prior work (37–39). Briefly, phosphorylation of the response regulator (RR06) by its cognate histidine kinase (HK06) leads to repression of *cbpA* expression. By contrast, dephosphorylation of RR06 leads to *cbpA* activation (Fig. 5B) (37–39). While this transcriptional mechanism is well established, it remains to be determined whether *TCS06* controls microcolony biofilm formation in a manner consistent with, and dependent on, its regulation of *cbpA* expression. Therefore, we sought to establish how *TCS06* affects microcolony biofilms via *cbpA* expression, using LFAB on a series of deletion and overexpression mutants of this locus.

In our screen, we found that a Tn-insertion mapping proximal to the response regulator *rr06* displayed a complete abrogation of microcolony biofilms at 24 hours (Fig. 4C, position n3), whereas a clone with insertion inside the histidine kinase *hk06* displayed increased microcolony biofilms and lower background cell density (Fig. 4C, position n1). Furthermore, *hk06* was also a hit in the previous transposon screen by Muñoz-Elías et al. (32). The directionality of the phenotypes in our screen was consistent with the hypothesis that *hk06* and *rr06* mutants affect transcriptional levels of *cbpA*, which, in turn, controls microcolony biofilm development. Therefore, we predicted that deletion of the *hk06* would increase *cbpA* expression (as RR06 is dephosphorylated in this background) and hence increase microcolony biofilm biomass, whereas deleting *rr06* would decrease *cbpA* expression and hence decrease microcolony biofilm biomass (37).

To test our predictions, we generated clean deletions of *hk06* and *rr06* in the parental D39Δ*cps* background and performed time-lapse imaging to examine their microcolony biofilms using LFAB (Fig. S9). The phenotypes of these mutants were all consistent with our predictions based on the existing model of *cbpA* regulation. Specifically, we found that deletion of *hk06*, which increases *cbpA* expression, led to greater microcolony biofilm biomass than the parental strain (Fig. 5D; Fig. S9). This increase in biomass was dependent on *cbpA* expression, as deletion of *cbpA* in the Δ*hk06* background decreased biomass to the same level as the Δ*cbpA* single deletion (Fig. 5D; Fig. S9). In contrast, we found that deletion of *rr06*, which decreases *cbpA* expression, led to reduced biofilm biomass, displaying an intermediate phenotype between the parental strain and Δ*cbpA* (Fig. 5E; Fig. S9). These results suggest that the absence of *rr06* does not completely abrogate *cbpA* gene expression. Moreover, overexpression of *cbpA* in the Δ*rr06* background rescued the reduced microcolony biomass phenotype, suggesting that the decrease in microcolony biomass in the Δ*rr06* strain is due to diminished *cbpA* expression (Fig. 5E; Fig. S9). Furthermore, deletion of both the response regulator and the histidine kinase (Δ*TCS06*), which does not influence *cbpA* levels (38), did not significantly change the biofilm phenotype (Fig. S10A through C). These data strengthen our finding that *cbpA* drives microcolony biofilm stability by demonstrating that regulatory changes that increase *cbpA* expression promote microcolony biofilms, while those that decrease *cbpA* expression result in reduced microcolony biofilms. Finally, we tested two more hits in this genomic region, a small putative peptide (*SPD_2021*) encoded upstream of *rr06*, and the ClpC protease, using directed deletions or single-nucleotide mutations. These phenocopied the wild-type strains, suggesting that biofilm development defects observed in the Tn-mutants could be caused by polar effects of these Tn-insertions (Fig. S10A through C).

In summary, we found that the absence of *cbpA* led to defects in microcolony biofilm formation, while high levels of *cbpA* increased microcolony biofilm size and definition. Furthermore, changes in the levels of phosphorylated RR06, which modulates levels of *cbpA*, also drove differences in microcolony biofilm phenotypes. Taken together, using LFAB, we established that *cbpA* expression levels are directly associated with microcolony biofilm morphology and can be modulated by the individual components of the upstream two-component system *TCS06*, strongly suggesting that factors that impact TCS06 phosphorylation levels *in vivo* modulate biofilm stability.

### The peptidoglycan hydrolase LytB alters the spatial distribution and morphology of microcolony biofilms

**Fig 6:**
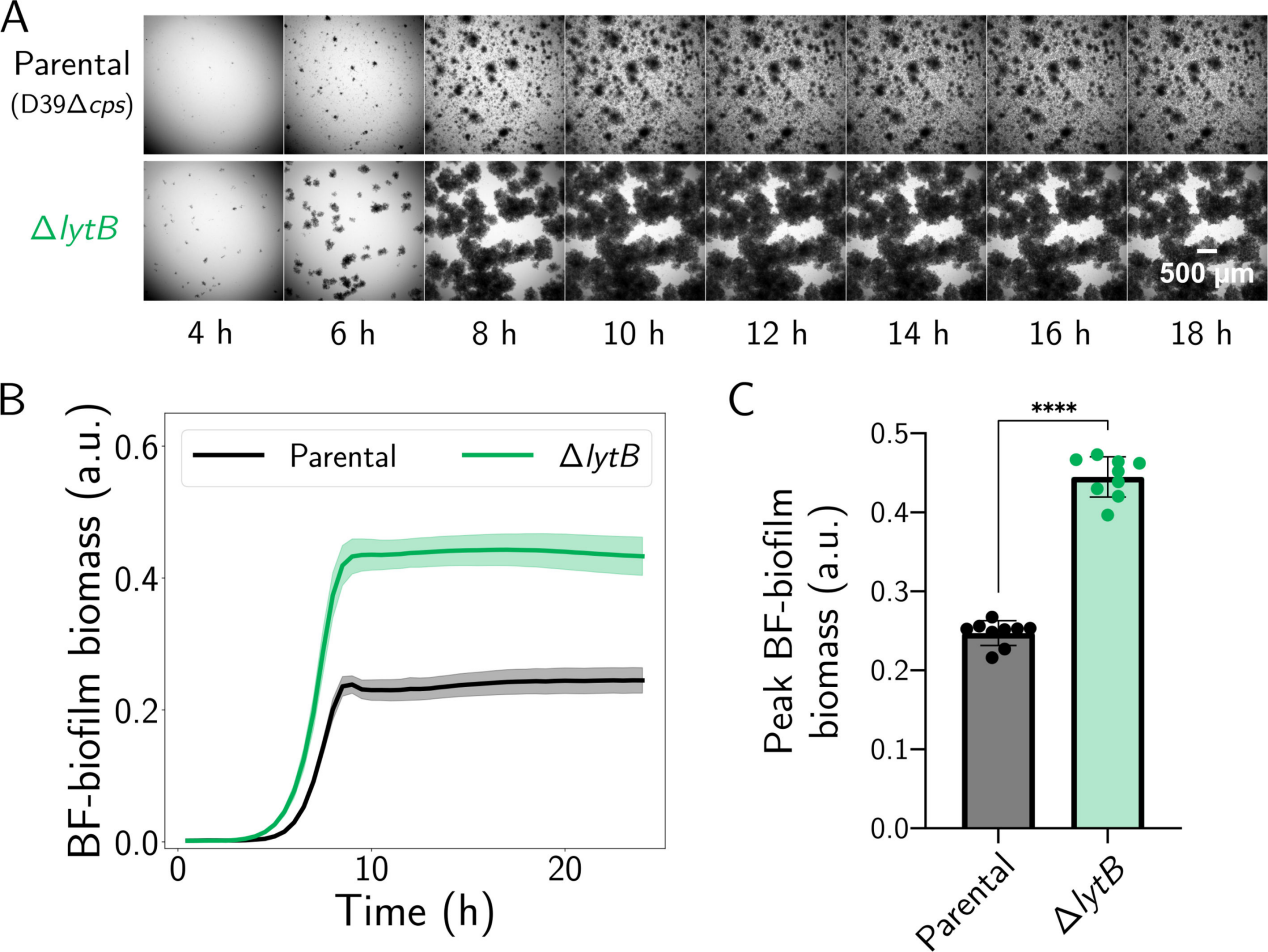
The peptidoglycan hydrolase LytB alters spatial distribution and morphology of microcolony biofilms. (**A**) Time-series brightfield images of the parental D39Δ*cps* strain and isogenic Δ*lytB* deletion strain, at the indicated time points. The scale bar is the same for all images and is indicated on the bottom right. (**B**) Time series of microcolony biofilm biomass for the above strains, as quantified by LFAB from 0 to 24 hours post-seeding at 30-min intervals. Lines show the mean, and shaded areas show the standard deviation. (**C**) Peak microcolony biofilm biomass for both strains. Each data point represents the peak biomass of an individual time series. *N* = 3 biological replicates, *n* = 3 technical replicates each. Student’s two-tailed *t*-test; ****P < 0.0001. BF: brightfield. a.u.: arbitrary units.

Our screen revealed multiple hits with increased biofilm biomass. One of the most dramatic from this set corresponded to an insertion into the choline-binding protein LytB (Fig. 4C, position f7). LytB is an N-acetylglucosaminidase that hydrolyzes peptidoglycan, an activity required for separation of daughter cells post-cell division, and therefore loss of LytB results in long chains of cells (65–67). Phenotypically, loss of LytB impairs attachment of *S. pneumoniae* to host epithelial cells and invasion of the lung epithelium (68, 69). To validate the hit from our screen, we generated a clean deletion mutant of *lytB* in the parental strain background and tested its microcolony biofilm phenotype (Fig. 6A; Movie S6). We found that the Δ*lytB* deletion strain displayed faster microcolony biofilm development and increased overall biomass than the parental strain (Fig. 6B and C). Moreover, we observed a dramatic decrease in interstitial non-microcolony cells in the mutant, compared to the parental strain (Fig. 6A). Thus, the peptidoglycan hydrolase LytB affects the spatial distribution, biomass, and morphology of *S. pneumoniae* microcolony biofilms.

## DISCUSSION

Given the limitations of existing methods, here, we asked whether label-free imaging could provide a broadly accessible means to quantify biofilm developmental dynamics. We developed LFAB, which uses low-magnification brightfield microscopy to exploit the intrinsic optical contrast of microcolony biofilms without the need for probes, stains, or genetic manipulation. In LFAB, bacterial populations are initiated at low seeding densities such that biofilm development from founder cells (or small, well-separated groups of cells) can be tracked before complete surface coverage occurs. Because confluence is not reached when microcolonies grow from low seeding densities, the observed biofilm outcomes reflect intrinsic founder-cell biofilm-forming capacity rather than homogeneous cell packing in dense populations. Thus, LFAB identifies microcolony rather than confluent biofilm morphologies. Furthermore, since brightfield microscopes are widely accessible to researchers across the world, LFAB can be readily implemented across laboratories and microbial systems. Notably, LFAB occupies a methodological space between CV staining and confocal microscopy: it combines the scalability and affordability of bulk assays with the ability to capture morphological features throughout biofilm development. Additionally, LFAB enables direct observation of biofilm lifecycle phases (formation, maturation, and dispersal) through time-lapse imaging—an advantage that we leveraged throughout this study, including in the screen for *S. pneumoniae* mutants (Fig. 4; Fig. S7). LFAB measurements strongly correlate with both CV and confocal benchmarks, underscoring the robustness of our method for the study of multiple species. The accompanying automated analysis pipeline makes the LFAB approach accessible for broad adoption in studies of microbial community behavior. Future work will aim to expand the scope of the LFAB approach to quantify the dynamics of planktonic sub-populations and the more fine-grained details of microcolony biofilm morphologies.

As a demonstration of the utility of LFAB, and to further characterize biofilm formation in a major human pathogen, we applied LFAB to *S. pneumoniae.* We characterized microcolony biofilms, which allows us to track the morphology and dynamics of formation, growth, and dispersal of these structures. We found that, consistent with previous studies, biofilm formation is negatively correlated with capsule production in this organism (19, 27, 29, 42–44). We then performed a high-content imaging screen, which identified 120 candidate genes impacting the biofilm lifecycle of *S. pneumoniae*. The predicted functions for these genes include the transport and metabolism of carbohydrates, the synthesis of the cell wall, autolysis, and cell adhesion.

We validated two surface choline-binding proteins, LytB (65–67) and CbpA (33–36), and the TCS components regulating the latter (37–39). We showed that while CbpA is not required in the early stages of microcolony biofilms, it plays a critical role in the maturation and stability of these communities. CbpA is the most abundant surface protein in *S. pneumoniae* (33) and is well characterized as an adhesin to host cells (33–36). The mechanisms by which CbpA influences biofilms in the absence of host factors remain to be defined. We hypothesize that it facilitates bacterial cell-cell adhesion, since late-stage maturation and stability of biofilms, which requires cell-cell adhesion, are compromised in the Δ*cbpA* mutant (Fig. 5C; Fig. S8). Like many surface proteins, CbpA displays a wide range of allelic variants, and future studies will test whether and how these variations influence cell-cell contacts, and consequently biofilm maturation and structure (70, 71). In contrast to CbpA, deletion of the peptidoglycan hydrolase LytB was associated with increased biomass and large microcolony biofilms, with the vast majority of cells in the field of view being concentrated within the biofilms (Fig. 6A). LytB promotes cell separation, such that defects in this protein lead to longer chains (65–67). We propose that increased chaining in the Δ*lytB* deletion mutant promotes adhesion, as well as an architecture that resists shear and traps cells and matrix. Alternatively, changes in *lytB* may affect biofilms indirectly by driving hyper-competence due to autocrine-like signaling within chains (72), in turn promoting changes in gene expression such as upregulation of BriC, a peptide linked to biofilm development (50). Interestingly, previous work has demonstrated that *lytB* promotes confluent biofilms (42, 73). This apparent mismatch is likely to result from the different physical features of microcolony biofilms versus confluent biofilms, both of which may be relevant to different aspects of infection. Overall, we present a list of candidate genes involved in *S. pneumoniae* microcolony biofilms, establish the role of LytB and CbpA in biofilm development, and demonstrate that LFAB serves as an effective tool to characterize *S. pneumoniae* biofilm determinants.

The screen revealed another cell surface virulence determinant, the sialidase enzyme neuraminidase A (*nanA*) (74). NanA contributes to colonization and biofilm development by cleaving sialic acid residues from host cells (47, 75). It also encodes a lectin-like domain that affords it additional adhesive properties (76). We found that *nanA*::*Tn* exhibited decreased microcolony biofilm biomass (Fig. 4C, position i10). Similarly, a previous study on confluent biofilms found that disruption of *nanB*, a gene encoding a second sialidase, also hindered biofilm development (32). The presence of NanA and CbpA, characterized as host-binding proteins, in our screen suggests these cell-surface proteins have additional biofilm-related functions that are independent of host cells.

A previous transposon screen for *S. pneumoniae* biofilm determinants, conducted using the CV assay by the Camilli lab (32), offers an opportunity to compare our findings to those obtained using traditional biofilm assays. The original study screened both encapsulated and unencapsulated strains seeded at high densities in the TIGR4 strain background (for which we did not measure appreciable microcolony biofilm formation). In the encapsulated strain, aside from capsule biosynthesis genes, this study solely identified *lytC* as a gene potentiating biofilms (32), which was also a hit in our screen. In the unencapsulated background, we identified 10 genes common to both sets: choline-binding protein A (33–36), its neighboring histidine kinase (37–39) and Clp protease (64), another choline-binding protein CbpF (77), a cardiolipin synthase, ribonuclease Y (78), a putative glycosyl hydrolase, the cell wall synthesis protein MurM (59–62), and two hypothetical proteins. In addition, we observed a mild biofilm reduction for an insertion in a neuraminidase gene *nanA*, and the Camilli lab’s study (32) yielded the other neuraminidase encoded by *S. pneumoniae*, *nanB* (79) as a hit. Together, we take the consistency between these screens as proof of principle for the LFAB approach and as a demonstration of common features for confluent and microcolony biofilms. Our screen also identified 110 unique hits. These include the cell wall peptidoglycan synthesis enzyme MurN (59), the mannitol transporter system, two surface-localized proteins (NanA [74] and Pbp2a [80]), the surface charge modifying protein DltA (81), multiple enzymes involved in carbohydrate processing (55, 56), and general metabolism (e.g., GalT, GalE, GlgA, PdxS, NrdH), several carbohydrate transporters in the ABC and phosphotransferase system (PTS) families (55), an Rgg/SHP cell-cell communication system Rgg1518 (82), the endonuclease EndA involved in DNA uptake (83), the DNA repair protein RadA (84), and numerous proteins with uncharacterized functions.

In summary, studies on *S. pneumoniae* biofilms have revealed that capsule, cell-cell communication, nutrient transporters, cell wall synthesis, and adhesins form the genetic toolkit that drives *S. pneumoniae* biofilms and likely chronic colonization. Yet, a clear playbook of when and how these components are combined to drive formation, maturation, and dispersal of *S. pneumoniae* remains to be determined. Furthermore, it is unclear whether there is one major pathway driving these processes or whether multiple different pathways achieve similar outcomes. Finally, questions remain as to whether microcolony biofilms or high-density confluent biofilms most closely model the biofilms associated with *S. pneumoniae* carriage, chronic middle ear infections, and heart disease. We propose that LFAB provides a valuable platform to continue studies of the molecular determinants and pathways that drive biofilm development across multiple human pathogens that form biofilms for colonization and/or infection of the host.

## MATERIALS AND METHODS

### Bacterial growth

All strains used in this work are reported in Table S2. *V. cholerae* and *P. fluorescens* strains were propagated on lysogeny broth (LB) plates supplemented with 1.5% agar or in liquid LB with shaking at 30°C. *P. aeruginosa* and *K. pneumoniae* strains were propagated on LB plates or in liquid LB with shaking at 37°C. *S. pneumoniae* strains were propagated on tryptic soy agar II (TSA-II) plates supplemented with 5% sheep blood (BD, BBL, New Jersey, USA; Ref. no. 221261) for overnight growth at 37°C in 5% CO_2_, or in Columbia broth (CB; Remel Inc.; Ref. no. R452972) statically at 37°C in 5% CO_2_.

For LFAB and confocal fluorescence measurements, *V. cholerae* strains were grown in M9 minimal medium containing dextrose and casamino acids (1× M9 salts, 100 µM CaCl_2_, 2 mM MgSO_4_, 0.5% [wt/vol] dextrose, 0.5% wt/vol casamino acids), *P. fluorescens* strains were grown in M63 minimal medium containing dextrose and casamino acids (1× M63 salts, 2 mM MgSO_4_, 0.5% [wt/vol] dextrose, 0.5% [wt/vol] casamino acids), *P. aeruginosa* strains were grown in M63 minimal medium containing arginine (1× M63 salts, 1 mM MgSO_4_, 0.4% [wt/vol] L-arginine), *K. pneumoniae* strains were grown in M9 minimal medium containing dextrose (1× M9 salts, 100 µM CaCl_2_, 1 mM MgSO_4_, 0.4% [wt/vol] dextrose), and *S. pneumoniae* strains were grown in CB. For *K. pneumoniae*, the M9 medium containing dextrose (but no additional salts) was chelated for 3 hours using Chelex 100 chelating resin (BioRad catalog no. 1421253). After filter sterilization, salts were added.

### Biofilm growth

For *V. cholerae*, *P. fluorescens*, and *P. aeruginosa*, unless otherwise indicated, strains were grown overnight in LB and subsequently back-diluted to ~10^4^ cells/mL in minimal medium. For *K. pneumoniae*, overnight LB cultures were washed 1× in PBS, then back-diluted to ~10^4^ cells/mL in minimal medium. For *S. pneumoniae* strains, cultures were grown overnight on TSA-II plates with 5% sheep blood at 37°C in 5% CO_2_. In the morning, cultures were inoculated into CB at OD_600_ ~0.02–0.03 and grown to OD_600_ = 0.05 (corresponding to ~10^7^ cells/mL). Cultures were then back-diluted in fresh CB to ~10^3^ cells/mL unless otherwise indicated. An amount of 200 µL of diluted cultures was subsequently grown statically in 96-well microtiter plates (Costar; Ref. no. 3370). For experiments pertaining to Fig. 2C, *V. cholerae*, *P. fluorescens*, and *P. aeruginosa* biofilms were grown for 24 hours and subsequently washed 6× in PBS media prior to imaging. The procedure was identical for *K. pneumoniae* but without wash steps to avoid biofilm disruption. For *S. pneumoniae*, the images were acquired at 10 hours post-seeding without washing to capture the peak stage of microcolony biofilm growth.

To compare biofilm growth across media conditions in *S. pneumoniae* (Fig. S5 and S6B), cells were grown as described above in 1× CB to OD_600_ = 0.05, that is, early log phase (corresponding to ~10^7^ cells/mL). Five milliliters of culture was centrifuged at 3,000 × *g* for 5 min, supernatant was removed, and cells were resuspended in an equal volume of the lowest concentration medium (0.25× CB). Centrifugation and resuspension in 0.25× CB were repeated two more times, and cells were then back-diluted into various medium concentrations to seed biofilms as described above. Colony-forming units (CFUs/mL) were determined to estimate cell number, and the dilution corresponding to ~10^3^ cells/mL was used for analysis.

### Microscopy

Brightfield time-lapse microscopy images were acquired at 30-min intervals at 30°C (*V. cholerae*, *P. fluorescens*, *P. aeruginosa*, and *K. pneumoniae*) or 37°C (*S. pneumoniae*) on an Agilent BioTek Cytation 1 imaging plate reader using either a 10× air objective (Olympus Plan Fluorite, NA 0.3) or a 4× air objective (Olympus Plan Fluorite, NA 0.13) driven by BioTek Gen5 (Version 3.12) software. The Cytation 1 was attached to an Agilent BioTek BioSpa 8 automated incubator (driven by BioSpa OnDemand software version 1.04), in which the samples were incubated and transferred at designated time points to the Cytation 1 by the robotic arm of the incubator. In Fig. S1, images were additionally acquired on an EVOS M5000 imaging system (Invitrogen, Thermo Fisher Scientific Inc.), using an EVOS Plan phase-contrast 10× air objective (0.30 NA/7.13 WD), or using a Nikon Ti-E inverted epifluorescence HCS imager, equipped with a Photometrics Evolve EMCCD camera and a 10× air objective, as indicated. In Fig. 6 and Fig. S6B, images were acquired on an Agilent BioTek Cytation 5 imaging plate reader using a 4× air objective (Olympus Plan Fluorite, NA 0.13) driven by BioTek Gen5 (Version 3.12) software. The Cytation 5 was also attached to an Agilent BioTek BioSpa 8 automated incubator (driven by BioSpa OnDemand software version 1.04), in which the samples were incubated, and transferred at designated time points to the Cytation 5 by the robotic arm of the incubator. Samples were grown in 96-well polystyrene microtiter plates (Costar; Ref. no. 3370) or in Mattek glass-bottom 96-well microtiter plates (MatTek Corporation; Part no. P96G-1.5-5-F). Spinning disc confocal images were acquired using a motorized Nikon Ti-2E stand outfitted with a CREST X-Light V3 spinning disk unit, a back-thinned sCMOS camera (Hamamatsu Orca Fusion BT), and a 20× air objective (Nikon Plan Apochromat, NA 0.75) driven by Nikon Elements software (Version 5.42.02). The source of illumination was an LDI-7 Laser Diode Illuminator (89-North). Cells were stained with 10 µM (or 20 µM for *S. pneumoniae*) of the lipophilic dye MM4-64 (AAT Bioquest) for 1 hour and were imaged with an excitation wavelength of 561 nm. *V. cholerae*, *P. fluorescens*, *P. aeruginosa*, and *K. pneumoniae* strains were grown in 18-well glass-bottom chamber slides (ibidi GmbH). *S. pneumoniae* strains were grown in a Mattek glass-bottom 96-well microtiter plate (MatTek Corporation; Part no. P96G-1.5-5-F). To ensure consistency in the comparisons between LFAB and other methodologies, we prepared samples in an identical manner between the methodologies being compared. For *V. cholerae*, *P. fluorescens*, *P. aeruginosa*, and *K. pneumoniae*, washing was performed in the comparison of LFAB with confocal, whereas for *S. pneumoniae*, washing was not performed due to the higher propensity of the biofilms to become dislodged.

To acquire an *I*_min_ image (optional shutter closed image for LFAB analysis; see below), an opaque black 96-well plate (Thermo Fisher Scientific; Catalog no. 237105) was placed in the plate reader. Images of five different wells were acquired, with an identical objective lens, light intensity, exposure time, and camera gain settings as used for the experiment. The images were averaged pixel-wise, and the averaged image was used as the *I*_min_ input to the LFAB pipeline.

### LFAB image analysis and web application

All codes for image analysis were written in the Julia programming language (v1.11.2) (85) and are publicly available on GitHub (https://github.com/BridgesLabCMU/Brightfield-biofilm-assay). All brightfield images were subjected to the same image analysis pipeline. A web application interface for performing image analysis can be used, and the instructions are provided on the GitHub page.

To generate a mask (i.e., a spatial indicator) of biofilms in a brightfield image or time series of images, a corresponding “background” image is generated through the application of a large-kernel Gaussian filter (default side length = 100 pixels in *x* and *y*; for *S. pneumoniae* and *K. pneumoniae*, we used side length = 200 pixels in *x* and *y* due to large biofilm sizes). The background image is then subtracted from the raw image, normalizing the local contrast within an image and, for time-lapse imaging, normalizing the background intensity across timepoints. At this point, time-lapses are registered (aligned) using the single-step discrete Fourier transform algorithm described by Guizar-Sicairos and colleagues to correct for frame-to-frame jitter (86). A small-kernel Gaussian filter (side length = 2 pixels in *x* and *y*) is then applied to control for intensity variation within biofilms, and a constant threshold is applied to generate a segmented mask of biofilms. In our study, the following thresholds were used for respective organisms: 0.03 (*S. pneumoniae* and *K. pneumoniae*) and 0.04 for all other species. If dust correction is selected (time-lapses only), all pixels in the mask that are 1’s at the beginning of the time-lapse and transition to 0’s at any point are taken to be dust and are set to 0 throughout. If closed-shutter and blank images (*I*_min_ and *I*_max_, respectively) are not supplied, the mask is directly applied, pixel-wise, to the raw image, and the average value in the resulting image yields the “biofilm biomass” value. All *S. pneumoniae* data, aside from those presented in Fig. 2C and S1A (for inter-species comparison), and Fig. S5 and S6B (to compare across different media) were analyzed in this manner, without supplying *I*_min_ and *I*_max_ images. If *I*_min_ and *I*_max_ images (or just *I*_min_ if the first image of a time-lapse is taken to be a blank image) are supplied, an optical density image is calculated as


$$I_{OD}=-\log_{10}\left(\frac{I_{raw}-I_{min}}{I_{max}-I_{min}}\right).$$

The mask is then applied, pixel-wise, to the optical density image, and the average value for all pixels in the resulting image yields a “biofilm biomass” value. For display purposes (in particular, for time-lapses, where the background intensity might change dramatically due to growth of planktonic cells), local contrast and global (temporal) background intensity are normalized using a local averaging filter based on integral arrays.

In general, we recommend comparisons only within the same well geometry. Furthermore, normalization to a biological control (e.g., an untreated wild type) is strongly recommended to control for non-optical sources of experimental variation.

### Confocal fluorescence microscopy image analysis

Confocal fluorescence images were acquired as described above. For each image, we reduced the contribution of Poisson shot noise in the images using the Denoise.ai module available in Nikon Elements software (Version 5.42.02). We then performed top-hat filtering of each *x-y* slice to reduce the contribution of out-of-focus light along the optical axis. A *z*-projection was computed and then blurred using a Gaussian filter (side length = 10 pixels). A bounded automatic threshold was then applied to the *z*-projection using Otsu’s method, generating a 2D mask. In effect, this procedure isolated regions in the *x-y* plane that contained biofilms (regions of locally greater thickness than the surroundings). Then, for each slice in the original tiled *z*-stack (corrected for anisotropy along the optical axis), a mask of cells was computed using a bounded automatic threshold, again using Otsu’s method. The *z*-projection mask was then applied to the cell mask, and the sum of the pixels in the resulting binary image yielded a value for the cell area occupied by biofilms. The sum of these areas over all slices yielded a total biofilm biomass for the 3D image.

### Crystal violet assay

For *S. pneumoniae*, biofilms were seeded at 10^3^ cells/mL into 12-well microtiter plates (VWR USA; catalog no. 10062-894) using CB as growth media and grown for 24 hours. At the endpoint, supernatant was removed, and biofilms were gently washed three times with PBS. Biofilms were then incubated with 0.1% CV solution for 10 min at room temperature. Excess stain was removed with three more PBS washes. CV was then quantified by solubilizing it in 70% ethanol and measuring its absorbance at 600 nm on the spectrophotometer. The protocol for *V. cholerae* was identical except that cultures were grown in M9, as described above, in 24-well plates (Costar, Corning Inc., USA; Ref. no. 3526). Readings were taken at 590 nm on the spectrophotometer.

### Bacterial construct generation and transformation

For generating *S. pneumoniae* mutants, we used a two-step transformation approach to generate clean deletions using the fluorescent removable antibiotic cassette (FRANC) containing spectinomycin-resistance *aad9*, sucrose-sensitivity *sacB*, and a fluorescent mCardinal marker (87). Briefly, for the first transformation, FRANC was inserted to replace the region desired for deletion. Colonies were screened for spectinomycin resistance and mCardinal fluorescence. In the second step, the FRANC was removed using a construct with only the two flanking regions. Colonies were selected for sucrose resistance and loss of fluorescence.

In brief, around 2 kb of flanking regions upstream and downstream of the gene/region of interest, and FRANC was amplified by PCR using Q5 2× Master Mix (NEB, USA). PCR fragments were verified by gel electrophoresis and subsequently cleaned up via the DNA Clean & Concentrator-5 kit (Zymo Research, USA). Fragment assembly was carried out using an in-house GIBSON assembly mix. All primers used for construct generation are listed in Table S3.

Strains were inoculated in CB and grown to an OD_600_ of 0.05. Then, 1 mL culture was incubated with the previously assembled DNA construct and 125 ng CSP-1 peptide (EMRLSKFFRDFILQRKK, GenScript, USA). After a 2-hour incubation period, cultures were plated on respective CB agar spectinomycin plates (100 µg/mL spectinomycin) or CB agar sucrose plates (10% [wt/vol] sucrose). After overnight incubation, resistant colonies were picked, regrown in CB media with 100 µg/mL spectinomycin or 10% sucrose (wt/vol), and frozen stocks were generated. Mutants were verified via PCR and Sanger sequencing.

### Isolation/generation of unencapsulated *S. pneumoniae* strains

The unencapsulated version of the SV36 strain was isolated as a spontaneous mutant in the lab. SV36, which expresses the type 3 capsule, forms visibly thick mucoid colonies on TSA + blood plates. Spontaneous mutants that do not express the capsule make visibly distinct non-mucoidal colonies. One such non-mucoidal colony was picked, propagated, and its genome was sequenced. We found a single nonsense mutation in the *cpsA* gene. This strain was used as the unencapsulated SV36 in this study.

The unencapsulated D39 was engineered by making a markerless gene deletion using the FRANC-based process described above. The deletion spanned multiple capsule genes (*cpsA-H* and *cpsT*) and mirrored the natural deletion found in the unencapsulated lab strain R6. This engineered strain was used as the unencapsulated D39 in this study.

### Transposon library generation and screening

The indexed transposon (Tn) library was prepared using genomic DNA from a pre-existing pooled Tn-mutant library in the D39 WT (encapsulated) background by transforming it into D39Δ*cps* and picking single colonies from a selection plate (88). Each colony was picked into an individual well of a 96-well plate containing Columbia broth (CB), grown for 8 hours at 37°C in 5% CO_2_, and frozen stocks were generated. The library consisted of 25 such 96-well plates; together they total 2,366 mutants. Each colony represents an independently isolated transposon insertion and is therefore expected to map to a unique genomic location. We sequenced a subset of these colonies, those with biofilm biomass trajectories that differ from the parental. In all sequenced isolates, the insertions were independent, although some mapped to the same operon or even the same gene, consistent with high selection at these loci (Table S1; Fig. 5A). Because the library was originally constructed for exploratory rather than saturation purposes, genome-wide coverage is incomplete, and not all ORFs may be represented.

To grow the library subsequently for screening, an autoclaved PCR plate was used as a “stamp” to inoculate each frozen stock plate into a fresh 96-well plate containing CB. These were grown for 8 hours at 37°C in 5% CO_2_, and a liquid-handling robot (Opentrons) was used to dilute down to 10^−5^ for seeding biofilms into another 96-well plate. The biofilms were grown for 24 hours and imaged at the endpoint on the Agilent BioTek Cytation 1 imaging plate reader using a 4× magnification objective.

### Identification of transposon insertion location by arbitrary PCR and Sanger sequencing

To identify the location of transposon inserts, a PCR was run using Q5 2× Master Mix (NEB, USA), with a forward primer inside the Tn sequence (P1) at 0.5 µM concentration, a random reverse primer mixture containing a defined overhang (adapter) sequence (P2) at 10× concentration of P1 (5 µM), and a cell suspension of the Tn-mutant as template. The following PCR conditions were used: 98°C for 30 s + (98°C for 15 s + 30°C for 30 s + 72°C for 30 s) × five cycles + (98°C for 15 s + 55°C for 30 s + 72°C for 30 s) × 30 cycles + 72°C for 2 min. The product of this first PCR was used as a template to run a second PCR (conditions: 98°C for 30 s + [98°C for 15 s + 55°C for 30 s + 72°C for 30 s] × 30 cycles + 72°C for 2 min), using another forward primer (P3) inside the Tn sequence (downstream of where P1 binds), and a reverse primer that binds to the overhang adapter sequence in P2. Following the second PCR, the amplified fragment was purified by the DNA Clean & Concentrator-5 kit (Zymo Research, USA). It was Sanger-sequenced using primer P3, by Genewiz from Azenta Life Sciences, USA. All primer sequences are provided in Table S3.

Upon receipt of the Sanger sequencing read, the portion of the read that matched the end of the Tn was trimmed. The trimmed read was used as a query to run nucleotide BLAST against the *S. pneumoniae* D39 genome on NCBI (RefSeq accession GCF_000014365.2). The region of best match (start coordinate of BLAST hit) was recorded as the insertion location of the transposon. The locus was inspected visually, the nearest gene in the RefSeq annotation was identified, and the gene IDs (new NCBI ID “SPD_RSxxxxx” and old ID “SPD_xxxx” were recorded. Corresponding R6 IDs “sprxxxx” and TIGR4 IDs “SP_xxxx” were identified using PneumoBrowse (52).

## Data Availability

The source data used to generate all main and supplemental figures in this work are available on KiltHub (https://doi.org/10.1184/R1/30238573).

## SUPPLEMENTAL MATERIAL

Supplemental material mbio.00448-26-s0001.docx Supplemental figures, movie legends, and tables.

Table S1 mbio.00448-26-s0002.xlsx Descriptions of hits from transposon mutant screen.

Movie S1 mbio.00448-26-s0003.avi Biofilm development in a *V. cholerae* strain carrying a chromosomal *Pbad-vpvC*^W240R^ construct, uninduced.

Movie S2 mbio.00448-26-s0004.avi Biofilm development in a *V. cholerae* strain carrying a chromosomal *Pbad-vpvC*^W240R^ construct, induced with 0.1% arabinose.

Movie S3 mbio.00448-26-s0005.avi Microcolony biofilm development in a D39Δ*cps* strain.

Movie S4 mbio.00448-26-s0006.avi Microcolony biofilm development in an SV36 *cps** (unencapsulated) strain.

Movie S5 mbio.00448-26-s0007.avi Microcolony biofilm development in a D39Δ*cps*Δ*cbpA* strain.

Movie S6 mbio.00448-26-s0008.avi Microcolony biofilm development in a D39Δ*cps*Δ*lytB* strain.

ASM does not own the copyrights to Supplemental Material that may be linked to, or accessed through, an article. The authors have granted ASM a non-exclusive, world-wide license to publish the Supplemental Material files. Please contact the corresponding author directly for reuse.
