# Supplementary material for: Label-free microscopy enables high-throughput identification of genes controlling biofilm development
Source: mBio. 2026 May 13;17(6):e00448-26. doi: 10.1128/mbio.00448-26 (PMC13251385; doi:10.1128/mbio.00448-26)
Supplement: Supplemental material — Supplemental figures, movie legends, and tables. [file mbio.00448-26-s0001.docx]

**Supplementary Material for:**

Label-free microscopy enables high-throughput identification of genes controlling biofilm development

M. R. Pratyush^1^*, Jojo A. Prentice^1^*, Rory A. Eutsey^1^, Irina V. Mikheyeva^1^, N. Luisa Hiller^1^^, Andrew A. Bridges^1^^

^1^Department of Biological Sciences, Carnegie Mellon University, Pittsburgh, PA 15213

^ Co-corresponding authors to whom correspondence should be addressed. Emails: [bridges@cmu.edu](mailto:bridges@cmu.edu) and [lhiller@andrew.cmu.edu](mailto:lhiller@andrew.cmu.edu)

* Authors contributed equally to this work

**Supplemental Figures**

Supplementary Figure 1

**
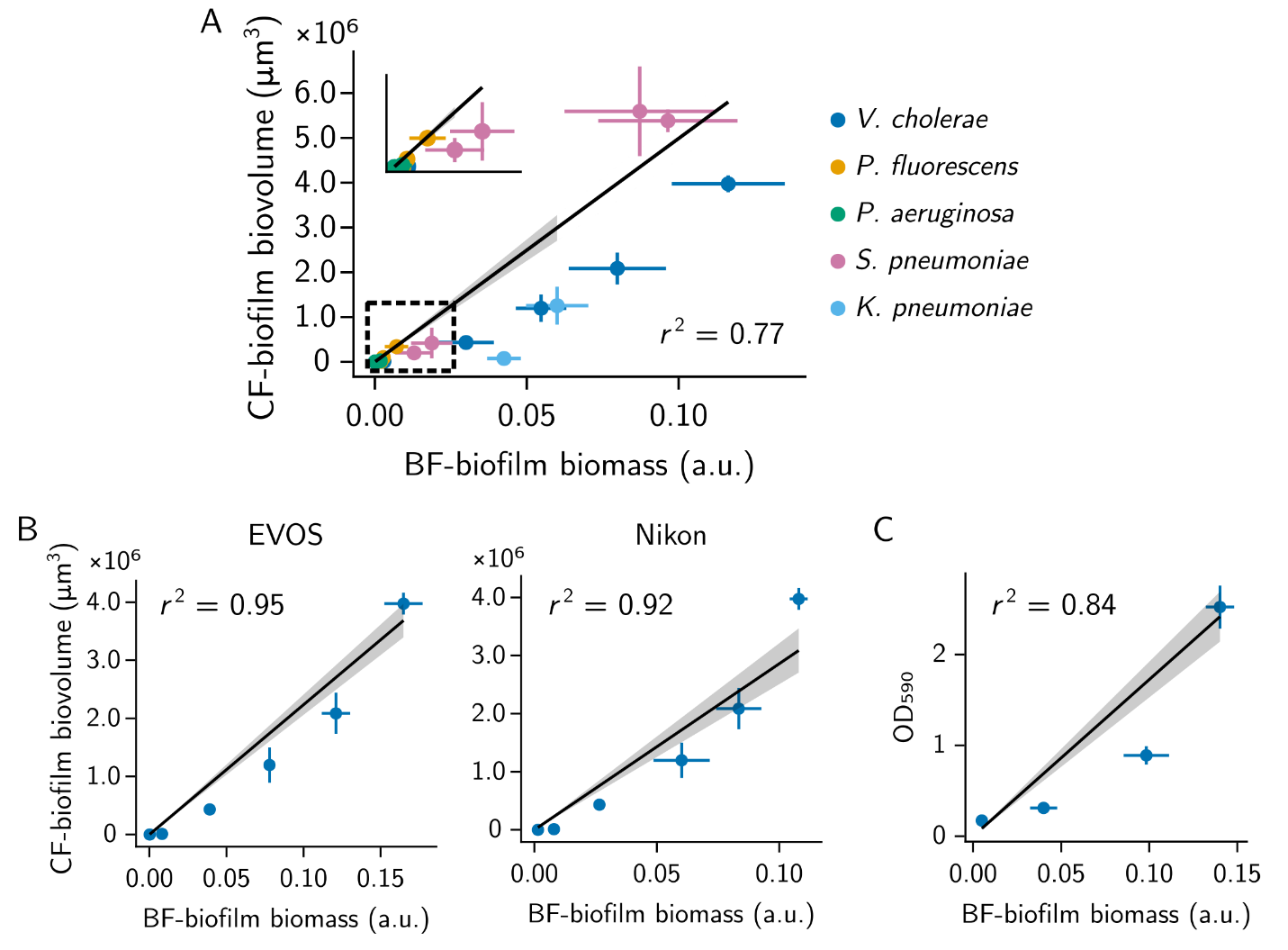
**

**Figure S1. LFAB correlations with confocal microscopy and crystal violet (CV) methods across microscopes and objectives.** In all panels, measurements were taken after 24 hours of growth. **(A)** Correlation of biofilm biomass calculated by brightfield microscopy (10x objective) on an Agilent Biotek Cytation 1 imaging plate reader to biofilm biovolume calculated by spinning-disc confocal microscopy (20x objective) for the indicated species. Each point represents a separate strain or biofilm induction condition. For all strains, parallel cultures were imaged by both confocal fluorescence microscopy and brightfield microscopy. Line represents the best-fit orthogonal distance regression to the data; r^2^ = 0.77 based on ordinary least squares. Inset displays the data points in the boxed region, with re-scaled axes. Error bars represent standard deviation. For brightfield biofilm biomass measurements, N = 3 biological with n = 3 technical replicates each. For confocal fluorescence biofilm biomass measurements, N = 3 biological replicates. **(B)** As in A but where brightfield biofilm biomass was measured on an EVOS (left) or Nikon (right) microscope for V. cholerae. Line represents the best-fit orthogonal distance regression to the data; r^2^ = 0.95 and r^2^ = 0.92, respectively, based on ordinary least squares. **(C)** As in A, B but a correlation between brightfield biofilm biomass and CV staining for V. cholerae (N = 3 biological replicates). Line represents the best-fit orthogonal distance regression to the data; r^2^ = 0.84 based on ordinary least squares. To achieve variation in biofilm biomass production in V. cholerae, a strain chromosomally encoding a Pbad-vpvC^W240R^ construct was used. This construct, when induced with arabinose, drives expression of a constitutively activated diguanylate cyclase, which in turn activates biofilm formation.^1^ The strain was induced with varying concentrations of arabinose. a.u.: arbitrary units. BF: brightfield. CF: confocal fluorescence.

Supplementary Figure 2

**
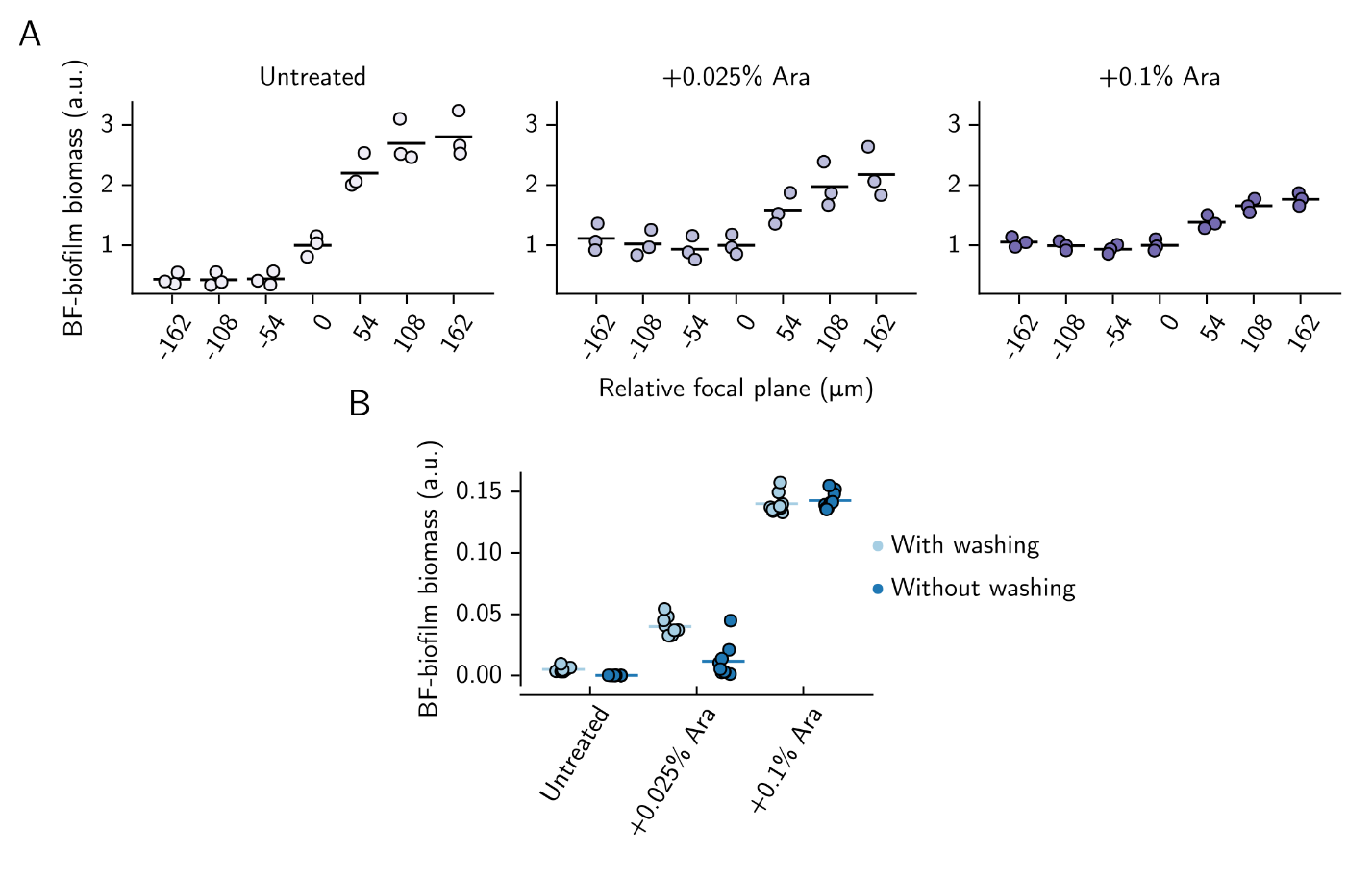
Figure S2. Technical considerations for the LFAB approach**. **(A)** Left: biofilm biomass of a V. cholerae strain carrying a chromosomal Pbad-vpvC^W240R^ construct^1^, without induction, as measured from brightfield images at the indicated focal planes. A focal plane of 0 represents an in-focus image. Data are normalized to the mean biofilm biomass of the in-focus images. Each point represents a replicate for N = 3 biological replicates. Middle, Right: as in the left panel for the same strain induced with the indicated arabinose concentrations. **(B)** Brightfield biofilm biomass of the same V. cholerae strain as in A, grown in the presence of the indicated concentrations of arabinose, and with or without an additional wash step as indicated. N = 3 biological replicates, n = 3 technical replicates each. Each point on the plot represents a technical replicate. Increasing levels of Pbad-vpvC^W240R^ induction result in increasing levels of biofilm biomass production.^1^ a.u.: arbitrary units. BF: brightfield. Ara: arabinose.

Supplementary Figure 3


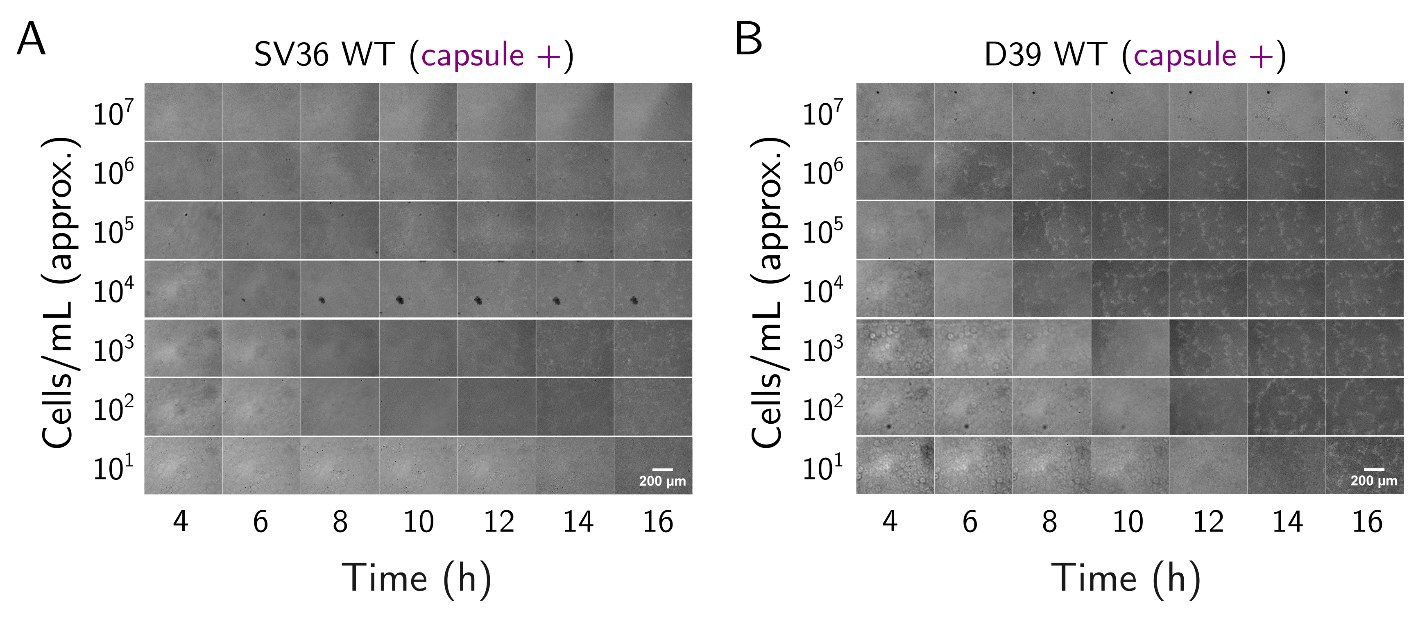


**Figure S3. Encapsulated WT strains SV36 and D39 form confluent biofilms at all seeding densities. (A)** Time series brightfield images of SV36 WT (encapsulated; type 3 capsule) at various initial cell seeding densities, showing highest cell inoculum at the top. **(B)** As in A, for D39 WT (encapsulated; type 2 capsule). All images were acquired at 10x magnification. Images are representative of N = 3 biological replicates with n = 3 technical replicates each. Scale bar is the same for all images, and is indicated on the bottom right of each panel.

Supplementary Figure 4


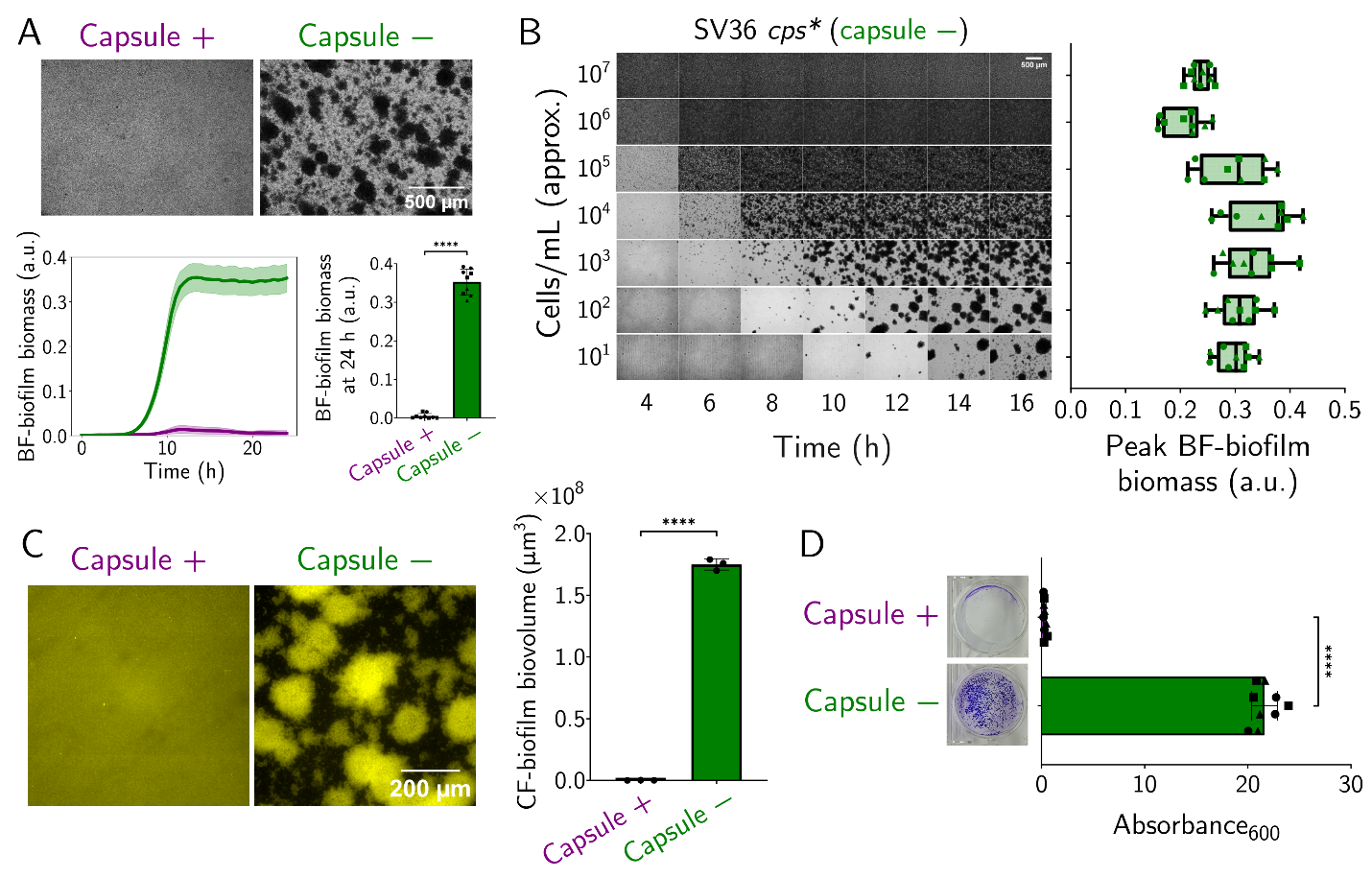


**Figure S4. Absence of capsule and low seeding density enable microcolony biofilm formation in S. pneumoniae (clinical strain SV36). (A)** Brightfield microscopy images (4x magnification) of clinical strain SV36 WT (type 3 capsule; left) and isogenic unencapsulated mutant (cps*; right) at 24 hours post seeding, grown from ~10^3^ cells/mL. Time series plot (bottom left) shows microcolony biofilm biomass for WT (purple, capsule +) and cps* (green, capsule –) strains, quantified by LFAB from 0-24 hours post seeding at 30 min intervals. Line shows mean and shaded region shows standard deviation. Bar plot (bottom right) shows BF-biofilm biomass at the 24-hour timepoint. **(B)** Time series brightfield images (left) of SV36 cps* at various initial seeding densities, showing highest seeding density at the top. Points on the boxplot (right) show the peak microcolony biofilm biomass for respective seeding densities across a 24-hour time series. Box plots show median and inter-quartile range, and whiskers show min and max values. **(C)** Confocal microscopy images (showing Z-stack sum projection) of WT (left) and cps* (right) SV36 strains. Biofilms were stained with 20 µM of the lipophilic dye MM4-64 and imaged at 24 hours post seeding at 20x magnification. Bar plot shows quantified cellular biovolume of microcolonies. **(D)** CV assay for SV36 WT and cps* at 24 hours post seeding. Biofilms were washed 3 times with phosphate buffered-saline (PBS), stained with 0.1% CV, and excess stain was removed with 3 more PBS washes. Images show representative wells after staining. CV was then quantified by solubilizing in 70% ethanol and measuring absorbance at 600 nm on the spectrophotometer. For LFAB and CV, N = 3 biological replicates with n = 3 technical replicates each; each point on the bar plot is a technical replicate, and each biological replicate is shown by a unique symbol. For confocal microscopy, N = 3 biological replicates; each point on the bar plot is a biological replicate Scale bars are as indicated. (C and D) Bars show mean and error bars show standard deviation. Student’s two-tailed t-test; **** p < 0.0001. BF: brightfield. a.u.: arbitrary units.

Supplementary Figure 5


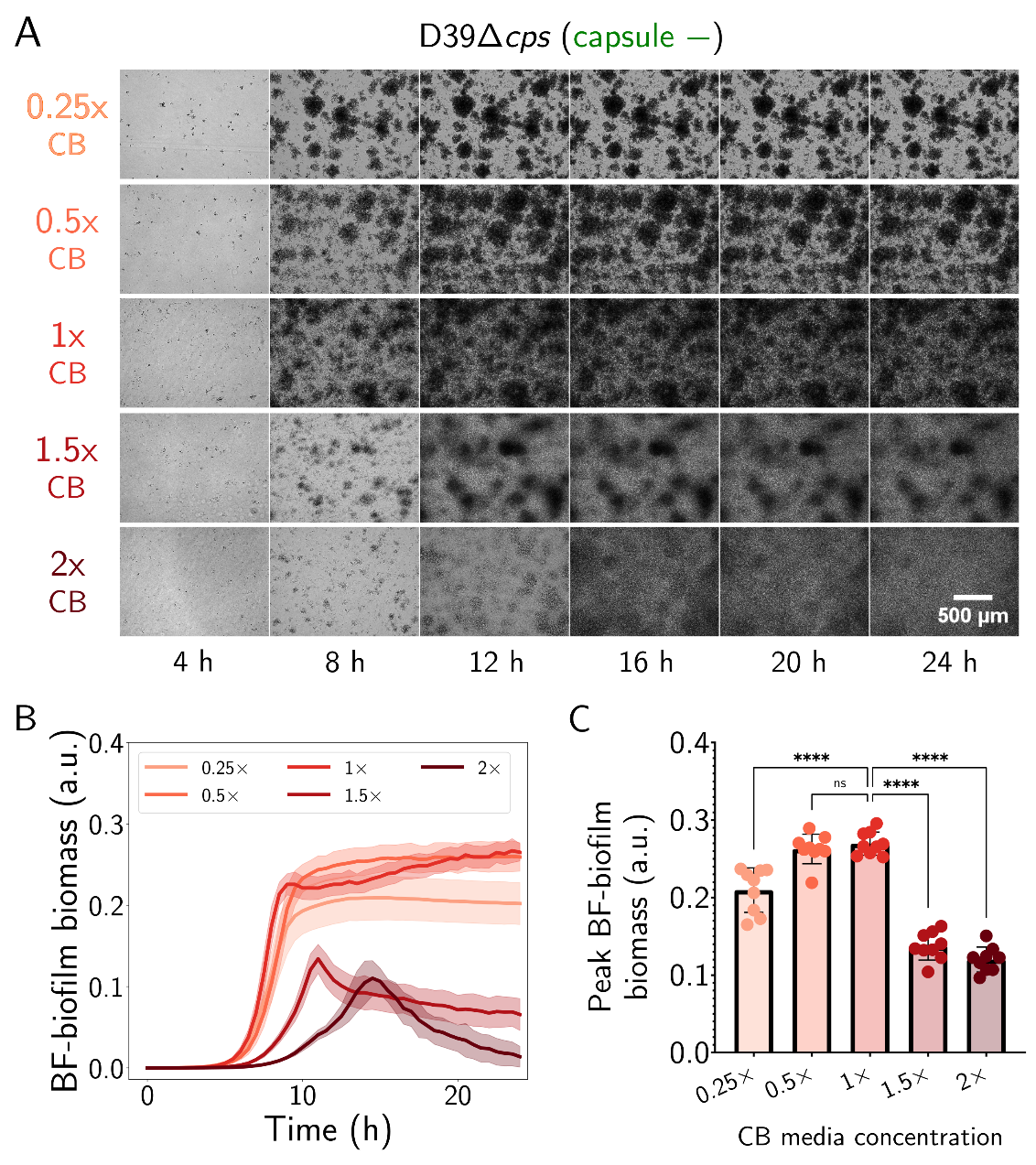


**Figure S5: Microcolony biofilm formation in unencapsulated D39Δcps** **is nutrient-limited in dilute media, but becomes impaired at high media concentrations.** **(A)** Time series brightfield images of unencapsulated D39Δcps at various concentrations of Columbia Broth (CB) growth medium. Scale bar is the same for all images and is indicated on the bottom right. **(B)** Time series plot shows microcolony biofilm biomass at various CB concentrations, quantified by LFAB from 0-24 hours post seeding at 30 min intervals. Line shows mean and shaded region shows standard deviation. **(C)** Peak microcolony biofilm biomass for each CB concentration. N = 3 biological replicates with n = 3 technical replicates each. In the bar plot, each data point represents the peak biomass of an individual time series. One way ANOVA, p < 0.0001. Dunnett’s multiple comparisons test (comparing each condition to 1x CB); **** p < 0.0001; ns: not significant. BF: brightfield. a.u.: arbitrary units.

Supplementary Figure 6


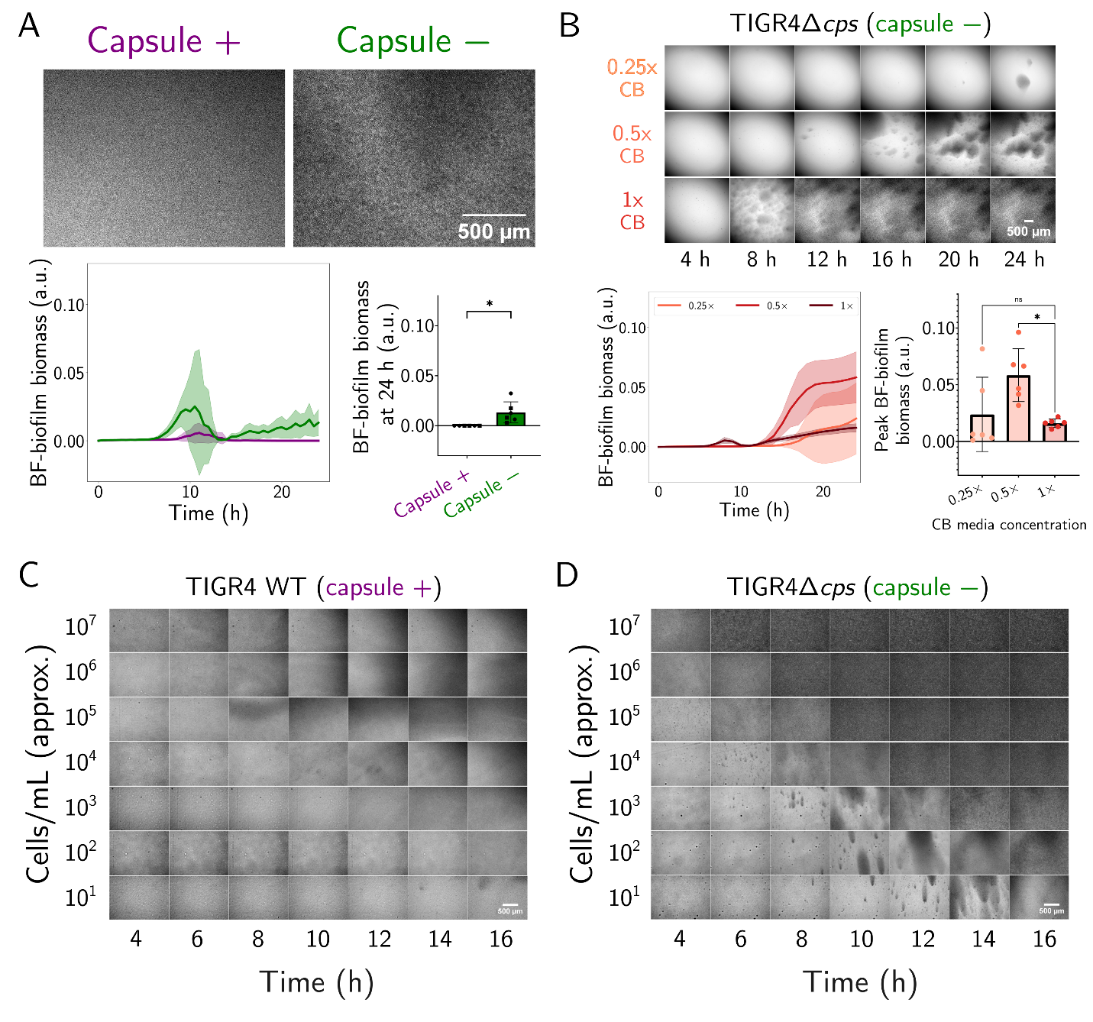


**Figure S6. Model strain TIGR4, when unencapsulated, produces microcolonies only in low-nutrient conditions.** The unencapsulated TIGR4 biofilms appeared thicker and more “grainy” in appearance at 24 hours post seeding compared to the encapsulated TIGR4 when grown in standard growth media (1x Columbia Broth; CB), which was reflected as a small difference in microcolony biofilm biomass when quantified by LFAB. Neither strain produced microcolony biofilms at any seeding density in 1x CB. However, when grown in diluted media, especially 0.5x CB, the unencapsulated TIGR4Δcps strain formed microcolony biofilms, but to a much lesser extent than the unencapsulated D39 and SV36. **(A)** Brightfield microscopy images (4x magnification) of model strain TIGR4 WT (type 2 capsule; left) and isogenic unencapsulated (TIGR4Δcps) mutant (right) at 24 hours post seeding, grown from ~10^3^ cells/mL in 1x CB. Time series plot (bottom left) shows microcolony biofilm biomass for TIGR4 WT (purple, capsule +) and Δcps (green, capsule –) strains, quantified by LFAB from 0-24 hours post seeding at 30 min intervals. Line shows mean and shaded region shows standard deviation. Bar plot (bottom right) shows BF-biofilm biomass at the 24-hour timepoint. **(B)** Time series brightfield images of unencapsulated TIGR4Δcps at various concentrations of Columbia Broth (CB) growth medium. Time series plot (bottom left) shows microcolony biofilm biomass at various CB concentrations, quantified by LFAB from 0-24 hours post seeding at 60 min intervals. Line shows mean and shaded region shows standard deviation. Bar plot (bottom right) shows peak microcolony biofilm biomass for each CB concentration. N = 2 biological replicates with n = 3 technical replicates each. In the bar plot, each data point represents the peak biomass of an individual time series. One way ANOVA, p < 0.05. Dunnett’s multiple comparisons test (comparing each condition to 1x CB); * p < 0.05; ns: not significant. **(C)** Time series brightfield images of TIGR4 WT (encapsulated) at various initial cell seeding densities in 1x CB, showing highest seeding density at the top. **(D)** As in C, but for TIGR4Δcps (unencapsulated). For panels A, C, and D, N = 3 biological replicates with n = 3 technical replicates each. Student’s two-tailed t-test; * p < 0.05. BF: brightfield. a.u.: arbitrary units. Scale bars are as indicated in each panel on the bottom right.

Supplementary Figure 7


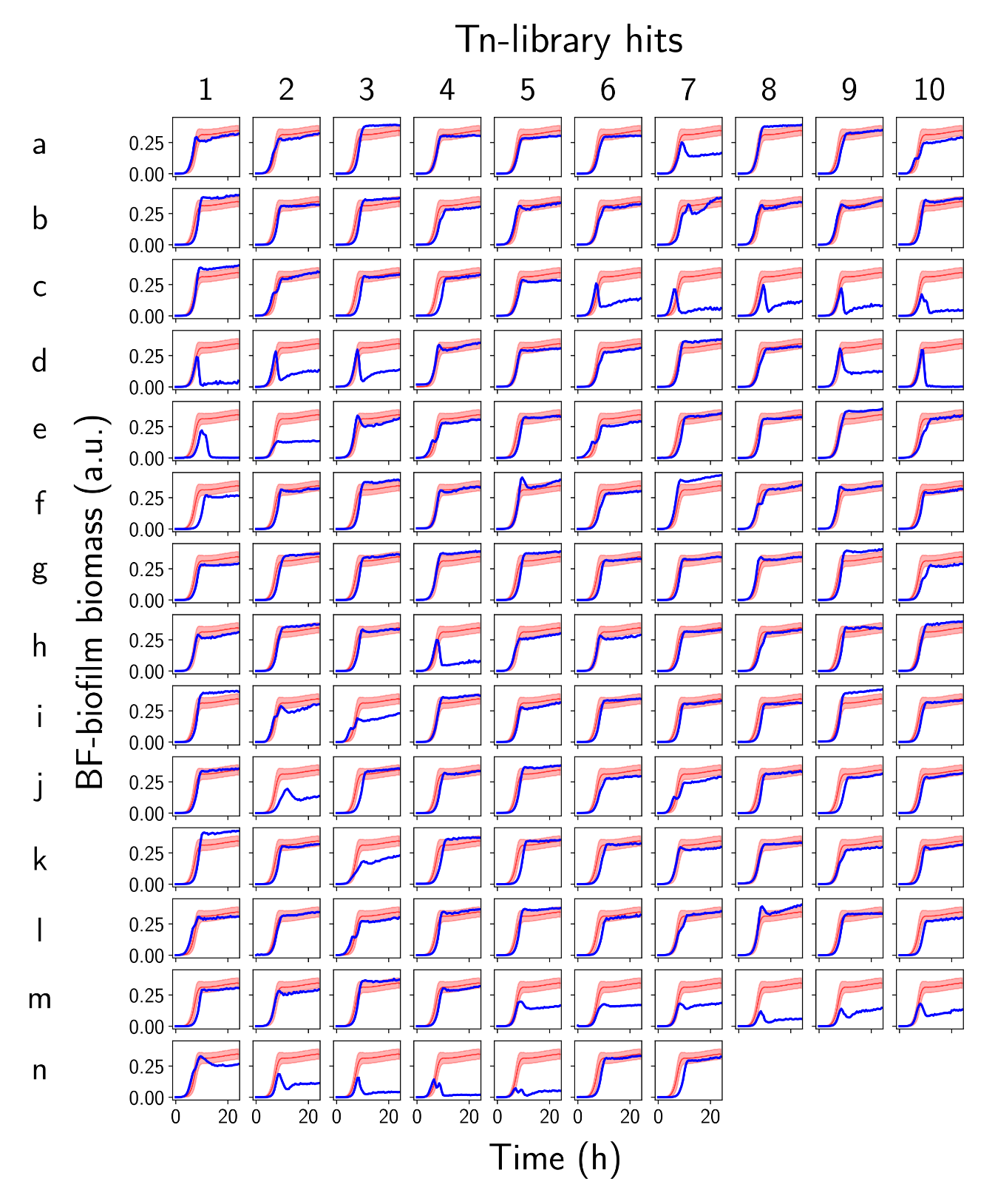


**Figure S7: Microcolony biofilm biomass time series trajectories for all hits from the Tn-mutant screen.** Each subplot shows the mean time series (blue) from N = 2 biological replicates of a Tn-mutant clone that was considered a hit in the screen. The figure shows all 137 Tn-mutant clones that were significantly different from the parental (66 below and 71 above the parental biofilm biomass threshold at endpoint; see **Fig. 4C**). The biomass time series trajectory of the parental strain (from N = 180 biological replicates) is shown in red on each subplot (red line shows mean and red shading shows standard deviation about the mean). Endpoint biofilm images for each clone are shown in **Fig. 4C** at the corresponding grid position. Details of all Tn-mutant clones are given in **Table S1**. Tn: transposon. BF: brightfield.

Supplementary Figure 8


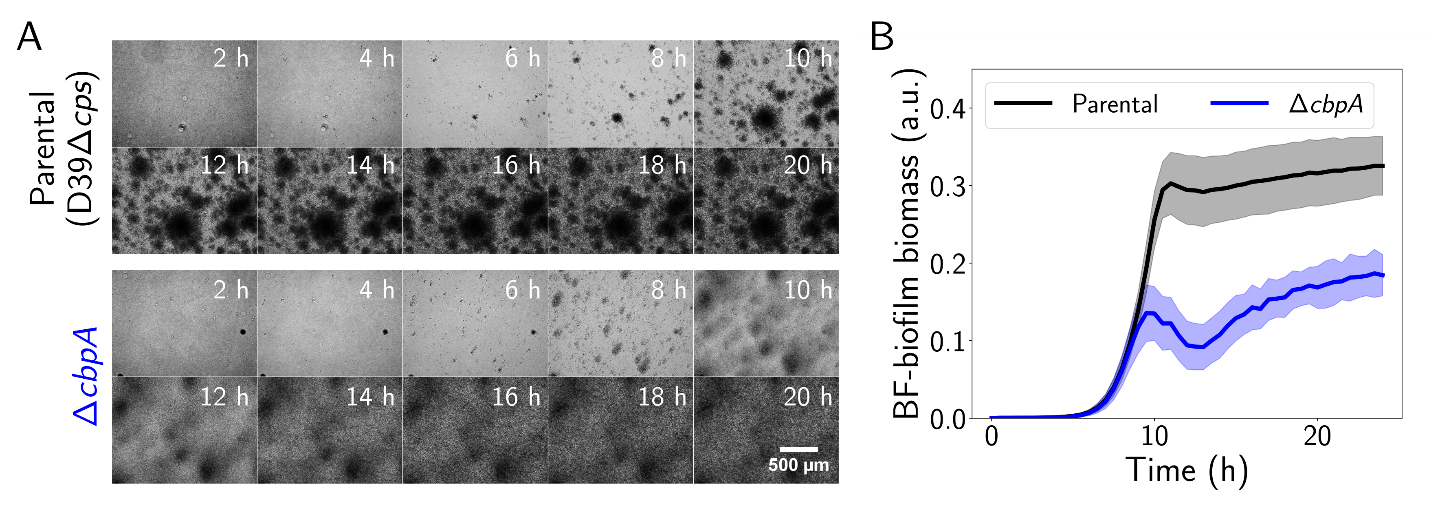


**Figure S8. Longitudinal analysis of parental (D39Δcps) and ΔcbpA biofilm development. (A)** Time series of biofilm development in the parental strain (D39Δcps; top) and ΔcbpA (bottom), showing images at indicated time points. Scale bar is the same for all images and is indicated on the bottom right. **(B)** LFAB quantification of the same strains, from 0-24 hours post seeding at 30 min intervals. Line shows mean and shaded region shows standard deviation. N = 3 biological replicates with n = 3 technical replicates each. BF: brightfield.

Supplementary Figure 9


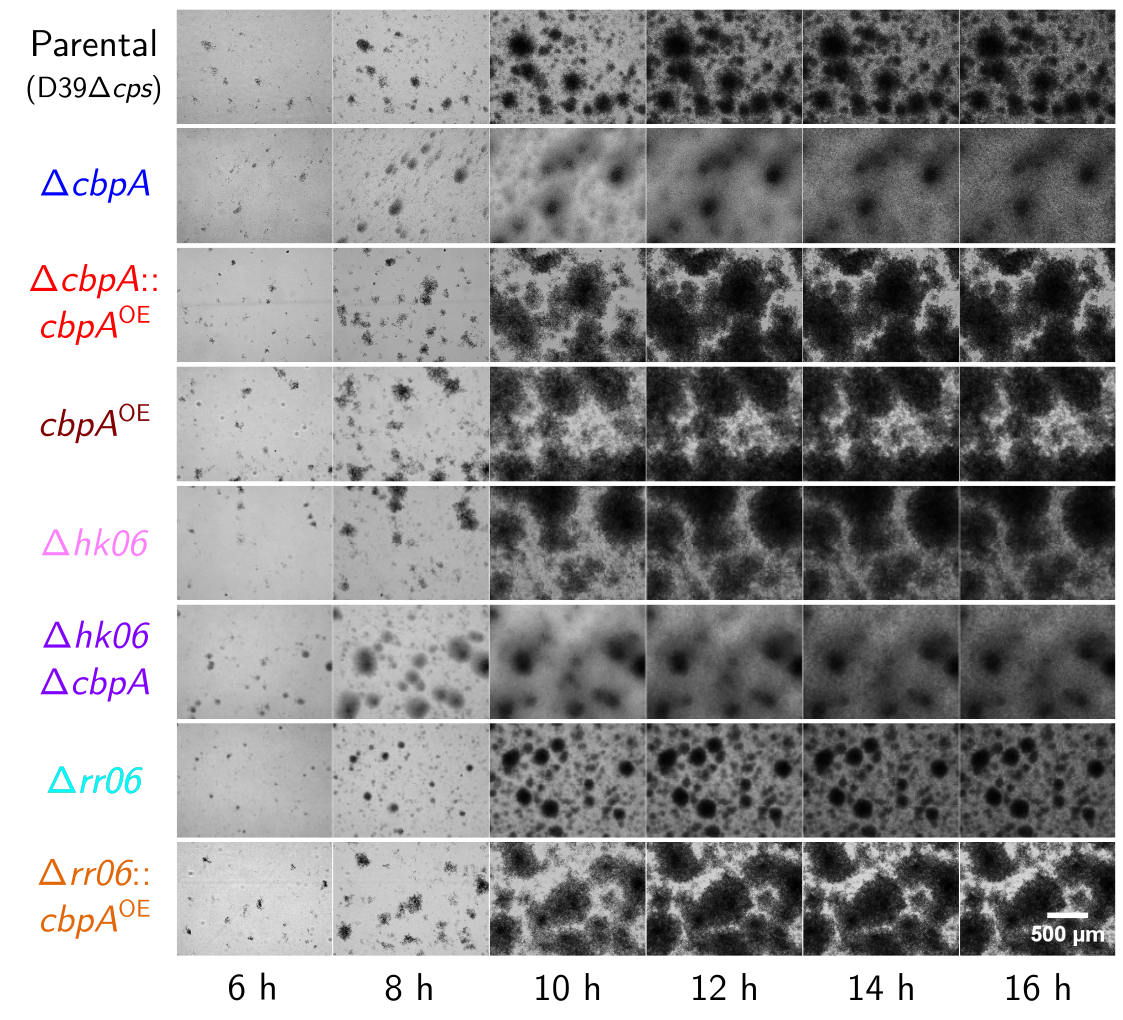


**Figure S9: Timelapse images of CbpA locus mutants.** Time series of biofilm development in the indicated mutant strains, showing images at the time points labelled below. Scale bar is the same for all images and is indicated on the bottom right. Images are representative of N = 3 to 10 biological replicates, with n = 3 technical replicates each.

Supplementary Figure 10

**
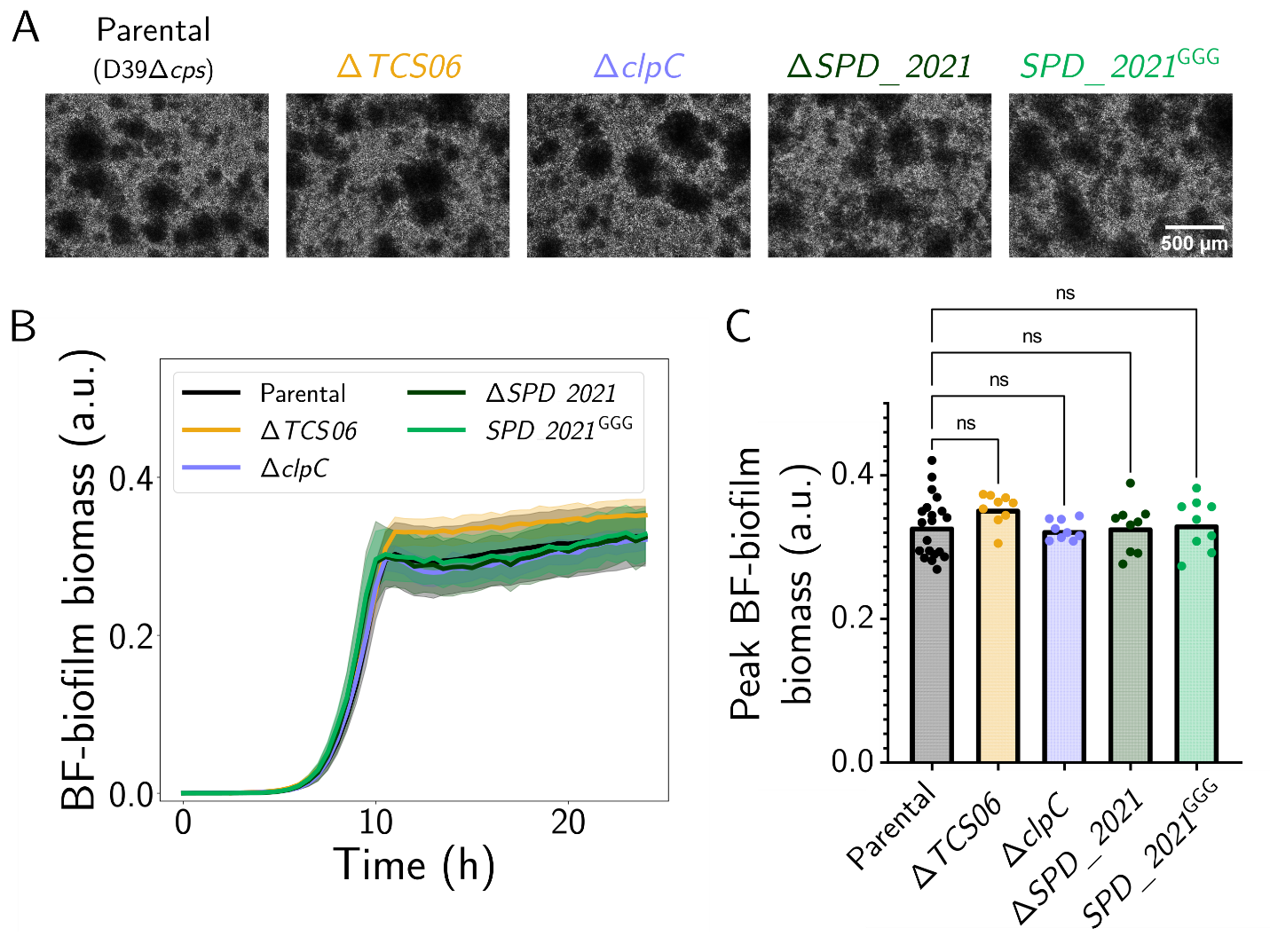
**

**Figure S10. Deletion of TCS06 (both hk06 and rr06), clpC, or SPD_2021, as well as mutating the start codon of SPD_2021 has no effect on microcolony biofilms.** Our Tn-screen revealed some additional mutations in the cbpA genomic locus that phenocopied ΔcbpA. Specifically, insertion mutations in (i) the region upstream of rr06 in the hypothetical peptide SPD_2021, and (ii) in the clp protease, ClpC. Here we show that deletion of these genes, as well as blocking translation of the predicted peptide, resembled the parental phenotype suggesting the biofilm defect may have been caused by polar effects of the transposon insertions. **(A)** Brightfield biofilm images (4x magnification) of the parental strain (D39Δcps), ΔTCS06, ΔclpC, ΔSPD_2021 and SPD_2021^GGG^ (start codon mutated to ‘GGG’) at 24 hours post-seeding. Scale bar is the same for all images and is shown on the bottom right. **(B)** Time series of microcolony biofilm biomass for the above strains, as quantified by LFAB. Lines show the mean of all biological and technical replicates, and shaded area shows standard deviation. **(C)** Peak microcolony biofilm biomass for each strain. For all strains, N = 3 to 10 biological replicates, with n = 3 technical replicates each. Each data point represents the peak biomass of an individual time series. One way ANOVA, p = 0.34 (not significant). Dunnett’s multiple comparisons test (comparing each strain to the parental); ns: not significant. BF: brightfield. a.u.: arbitrary units.

**Supplementary Movies (provided as separate AVI files)**

Supplementary Movie S1: Biofilm development visualized by brightfield microscopy images captured at 30 min intervals over 20 hours of growth (10x magnification) in a V. cholerae strain carrying a chromosomal Pbad-vpvC^W240R^ construct^1^, uninduced (therefore mimicking a wild-type V. cholerae). Scale bar and timestamps as shown.

Supplementary Movie S2: Biofilm development visualized by brightfield microscopy images captured at 30 min intervals over 23 hours of growth (10x magnification) in a V. cholerae strain carrying a chromosomal Pbad-vpvC^W240R^ construct^1^, induced with 0.1% arabinose. Scale bar and timestamps as shown.

Supplementary Movie S3: Microcolony biofilm development in a D39Δcps strain, visualized by brightfield microscopy images captured at 30 min intervals over 24 hours of growth (4x magnification). Scale bar and timestamps as shown.

Supplementary Movie S4: Microcolony biofilm development in an SV36 cps* (unencapsulated) strain, visualized by brightfield microscopy images captured at 30 min intervals over 24 hours of growth (4x magnification). Scale bar and timestamps as shown.

Supplementary Movie S5: Microcolony biofilm development in a D39ΔcpsΔcbpA strain, visualized by brightfield microscopy images captured at 30 min intervals over 24 hours of growth (4x magnification). Scale bar and timestamps as shown.

Supplementary Movie S6: Microcolony biofilm development in a D39ΔcpsΔlytB strain, visualized by brightfield microscopy images captured at 30 min intervals over 24 hours of growth (4x magnification in Agilent Biotek Cytation 5). Note that the Cytation 5 has a larger field of view than the Cytation 1, on which all other supplementary movies were captured. Scale bar and timestamps as shown.

**Supplementary Tables**

Supplementary Table S1: Descriptions of hits from transposon mutant screen, showing all mutants that were significantly different from the parental (**Fig. 4C** shows the corresponding images and **Fig. S7** shows the corresponding biomass time series)

Table provided as a separate Excel spreadsheet:

Supplementary_table_S1_Tn-screen_hits_details.xlsx

Supplementary Table 2: List of strains used in this study

| **Species** | **Strain** | **Description** | **Source** | **Notes** |
| --- | --- | --- | --- | --- |
| Streptococcus pneumoniae | D39 WT | Model strain D39, serotype 2 capsule (NCBI RefSeq ID GCF_000014365.2) | Hiller lab inventory | Originally received from Hasan Yesilkaya lab |
| Streptococcus pneumoniae | SV36 WT | Clinical isolate SV36, serotype 3 capsule (NCBI RefSeq ID GCF_000256645.1) | Hiller lab inventory | Originally received from Alexander Tomasz lab |
| Streptococcus pneumoniae | D39Δcps (Parental) | Unencapsulated D39, bearing a markless 7.5 kb deletion in the cps locus (spanning cpsA-H and cpsT) identical to the region deleted in strain R6. | This study | This is the parental strain for the transposon mutant library |
| Streptococcus pneumoniae | SV36 cps* | Unencapsulated SV36, isolated as a spontaneous mutant bearing a single nonsense mutation in the cpsA gene. | This study |  |
| Streptococcus pneumoniae | TIGR4 WT | Model strain TIGR4, serotype 4 capsule (NCBI RefSeq ID GCF_000006885.1) | Orihuela lab | Carlos Orihuela lab at University of Alabama at Birmingham^2^ |
| Streptococcus pneumoniae | TIGR4Δcps | Unencapsulated TIGR4, with a deletion of the entire capsule locus. | Orihuela lab | Carlos Orihuela lab at University of Alabama at Birmingham^2^ |
| Streptococcus pneumoniae | D39ΔcpsΔcbpA | Markerless deletion ΔcbpA (SPD_RS10670) in the D39Δcps background | This study |  |
| Streptococcus pneumoniae | D39ΔcpsΔcbpA::cbpA^OE^ | cbpA expression driven by run-off transcription from Spec cassette driven by amiA-F gene promoter, in the bgaA region. Made as a cbpA complement in the D39ΔcpsΔcbpA strain. | This study |  |
| Streptococcus pneumoniae | D39Δcps cbpA^OE^ | cbpA expression driven by run-off transcription from Spec cassette driven by amiA-F gene promoter, in the bgaA region. Made in the D39Δcps background. | This study |  |
| Streptococcus pneumoniae | D39ΔcpsΔhk06 | Markerless deletion Δhk06 (SPD_RS10680) in the D39Δcps background | This study |  |
| Streptococcus pneumoniae | D39ΔcpsΔhk06ΔcbpA | Markerless deletion ΔcbpA (SPD_RS10670) in D39ΔcpsΔhk06 background | This study |  |
| Streptococcus pneumoniae | D39ΔcpsΔrr06 | Markerless deletion Δrr06 (SPD_RS10685) in the D39Δcps background | This study |  |
| Streptococcus pneumoniae | D39ΔcpsΔrr06::cbpA^OE^ | cbpA expression driven by run-off transcription from Spec cassette driven by amiA-F gene promoter, in the bgaA region. Made in the D39ΔcpsΔrr06 strain. | This study |  |
| Streptococcus pneumoniae | D39ΔcpsΔhk06Δrr06 (ΔTCS06) | Markerless deletion of two component system 6 (ΔTCS06), i.e., double gene deletion Δhk06 & Δrr06 (SPD_RS10680, SPD_RS10685 respectively) in the D39Δcps background. | This study |  |
| Streptococcus pneumoniae | D39ΔcpsΔclpC | Markerless deletion ΔclpC (SPD_RS10700) in the D39Δcps background | This study |  |
| Streptococcus pneumoniae | D39ΔcpsΔSPD_2021 | Markerless deletion ΔSPD_2021 (a short ORF between SPD_RS10700 and SPD_RS10685) in the D39Δcps background | This study |  |
| Streptococcus pneumoniae | D39Δcps SPD_2021^GGG^ | Start codon of SPD_2021 (a short ORF between SPD_RS10700 and SPD_RS10685) was mutated to GGG, in the D39Δcps background | This study |  |
| Streptococcus pneumoniae | D39ΔcpsΔlytB | Markerless deletion ΔlytB (SPD_RS04590) in the D39Δcps background | This study |  |
| Vibrio cholerae | ΔvpsL | Strain C6706str2 | Bridges lab inventory |  |
| Vibrio cholerae | ΔVc1807::Pbad-vpvC^W240R^ | Strain C6706str2, encodes a kanamycin resistance cassette at Vc1807 | Bridges lab inventory |  |
| Pseudomonas fluorescens | SBW25 WT | - | Cooper lab inventory | Vaughn Cooper lab at University of Pittsburgh^3^ |
| Pseudomonas fluorescens | SBW25ΔwspF | - | Cooper lab inventory | Vaughn Cooper lab at University of Pittsburgh^3^ |
| Pseudomonas fluorescens | SBW25ΔwssA-J | - | Cooper lab inventory | Vaughn Cooper lab at University of Pittsburgh^3^ |
| Pseudomonas aeruginosa | PAO1 WT | - | Armbruster lab inventory | Catherine Armbruster lab at Carnegie Mellon University^4^ |
| Pseudomonas aeruginosa | PAO1ΔwspF | - | Armbruster lab inventory | Catherine Armbruster lab at Carnegie Mellon University^4^ |
| Klebsiella pneumoniae | KPPR1 WT | - | Mike lab inventory | Laura Mike lab at University of Pittsburgh^5,6^ |
| Klebsiella pneumoniae | KPPR1ΔwcaJ | - | Mike lab inventory | Laura Mike lab at University of Pittsburgh^5,6^ |

Supplementary Table 3: List of primers used in this study

| **Primer ID** | **Primer name** | **Sequence (5'-->3'): capital letters are annealing regions and small letters are overhangs meant for fragment assembly** |
| --- | --- | --- |
| SC241 | cps R6del F1 FWD | GCGGCCAATCAGGTTCAG |
| SC242 | cps R6del F1 IN REV | gtaactgggcAAGGTGTGAATGGACGAATC |
| SC243 | cps R6del Franc FWD | ttcacaccttGCCCAGTTACCAAATACAG |
| SC244 | cps R6del Franc REV | taatataaatCCTGTCTTTGGGCAGATAAAG |
| SC245 | cps R6del F2 IN FWD | caaagacaggATTTATATTATTTCCAATATTTGTTTATTATATAATAC |
| SC246 | cps R6del F2 REV | CCCAATAACTAACGCTCC |
| SC247 | cps R6del F1 OUT REV | taatataaatAAGGTGTGAATGGACGAATC |
| SC248 | cps R6del F2 OUT FWD | ttcacaccttATTTATATTATTTCCAATATTTGTTTATTATATAATAC |
| PMR60 | D39_cbpA-F1_Fwd | TCAAACCCAAGCTAGCTC |
| PMR61 | D39_cbpA-F1_in_Rev | gtaactgggcATGGTGAATGGGTAAACTAAAC |
| PMR62 | D39_cbpA-Franc_in_Fwd | cccattcaccGCCCAGTTACCAAATACAG |
| PMR63 | D39_cbpA-Franc_in_Rev | aaagcgaaagCCTGTCTTTGGGCAGATAAAG |
| PMR64 | D39_cbpA-F2_in_Fwd | caaagacaggCTTTCGCTTTTTGATGCAAAC |
| PMR65 | D39_cbpA-F2_Rev | TCTAGGTGGTGTTGGTCTAG |
| PMR66 | D39_cbpA-F1_out_Rev | aaagcgaaagATGGTGAATGGGTAAACTAAAC |
| PMR67 | D39_cbpA-F2_out_Fwd | cccattcaccCTTTCGCTTTTTGATGCAAAC |
| PMR68 | D39_hk06-F1_Fwd | CTTCAGCTTGTTGATCATC |
| PMR69 | D39_hk06-F1_in_Rev | gtaactgggcCACGGTATGGAATTTAAGATTAG |
| PMR70 | D39_hk06-Franc_in_Fwd | ccataccgtgGCCCAGTTACCAAATACAG |
| PMR71 | D39_hk06-Franc_in_Rev | gcttatgataCCTGTCTTTGGGCAGATAAAG |
| PMR72 | D39_hk06-F2_in_Fwd | caaagacaggTATCATAAGCTAATCTTATACCC |
| PMR73 | D39_hk06-F2_Rev | CCCACCCAGATATCTTTAATG |
| PMR74 | D39-hk06-F1_out_Rev | gcttatgataCACGGTATGGAATTTAAGATTAG |
| PMR75 | D39_hk06-F2_out_Fwd | ccataccgtgTATCATAAGCTAATCTTATACCC |
| PMR76 | D39_hk06-rr06-Franc_in_Rev | agggagagagCCTGTCTTTGGGCAGATAAAG |
| PMR77 | D39_hk06-rr06-F2_in_Fwd | caaagacaggCTCTCTCCCTTTCTACTAC |
| PMR78 | D39_hk06-rr06-F2_Rev | TCACAGATGAAGCGGTTG |
| PMR79 | D39_hk06-rr06-F1_out_Rev | agggagagagCACGGTATGGAATTTAAGATTAG |
| PMR80 | D39_hk06-rr06-F2_out_Fwd | ccataccgtgCTCTCTCCCTTTCTACTAC |
| PMR89 | D39_cbpA_Fwd_seq | AAACGTCGCAGTTCGTGAATTCC |
| PMR90 | D39_cbpA_Rev_seq | GGACAGTGAAGCTTATGCTTGTC |
| PMR91 | D39_hk06_Fwd_seq / D39_rr06-F1_Fwd / D39_SPD2021-clpC-ctsR-F1_Fwd | ATTGTCTTGTGCACCATTGCC |
| PMR92 | D39_hk06_Rev_seq | GCGATATCAGGATGTAACAGTGG |
| PMR93 | D39_hk06-rr06_Rev_seq | ATGTCAAATCCGTTCTAGCTGG |
| PMR94 | D39_rr06-F1_in_Rev | gtaactgggcGTTGGGTATAAGATTAGCTTATG |
| PMR95 | D39_rr06-Franc_in_Fwd | tatacccaacGCCCAGTTACCAAATACAG |
| PMR96 | D39_rr06-F1_out_Rev | agggagagagGTTGGGTATAAGATTAGCTTATG |
| PMR97 | D39_rr06-F2_out_Fwd | tatacccaacCTCTCTCCCTTTCTACTAC |
| PMR98 | D39_rr06_Fwd_seq | ATATCCAAGTCATGCACAAGAGG |
| PMR105 | D39_SPD2021-clpC-ctsR-F1_in_Rev | gtaactgggcAGATGTATGGAGTTCTGG |
| PMR106 | D39_SPD2021-clpC-ctsR-Franc_in_Fwd | ccatacatctGCCCAGTTACCAAATACAG |
| PMR107 | D39_SPD2021-clpC-ctsR-Franc_in_Rev | gaattaggctCCTGTCTTTGGGCAGATAAAG |
| PMR108 | D39_SPD2021-clpC-ctsR-F2_in_Fwd | caaagacaggAGCCTAATTCTGCCAAGATTTG |
| PMR109 | D39_SPD2021-clpC-ctsR-F2_Rev | TTCAGTATGATACCATGTTTGCC |
| PMR110 | D39_clpC-F1_out_Rev / D39_SPD2021_Rev_seq | taagatgaacATTGGTGTCAAAGCAGGC |
| PMR111 | D39_clpC-F2_out_Fwd | tgacaccaatGTTCATCTTACTTCCCTTTTC |
| PMR112 | D39_SPD2021-F1_out_Rev | actctccgtcTCCGTTCTAGCTGGTATTTG |
| PMR113 | D39_SPD2021-F2_out_Fwd | ctagaacggaGACGGAGAGTAATAAACATATG |
| PMR114 | D39_SPD2021-GGG-F1_out_Rev | aattcatgggTTTATTACTCTCCGTCGTATTTG |
| PMR115 | D39_SPD2021-GGG-F2_out_Fwd | gagtaataaacccATGAATTTTGTGTTGAATGG |
| R387 | D39_cbpA-comp-in-bgaA_F1_Fwd / D39_bgaA_Spec-ins_Fwd_1_seq | GGACCATGTCTTTAATGATTC |
| PMR116 | D39_cbpA-comp-in-bgaA_F1_Rev | tttccttctaTCCTCCAGATATGGATCC |
| PMR117 | D39_cbpA-comp-in-bgaA_cbpA_Fwd | atctggaggaTAGAAGGAAATAAACATGTTTG |
| PMR118 | D39_cbpA-comp-in-bgaA_cbpA_Rev | ccattacatcGGTTTAGTTTACCCATTCAC |
| PMR119 | D39_cbpA-comp-in-bgaA_F2_Fwd | aaactaaaccGATGTAATGGGTATGGATG |
| R392 | D39_cbpA-comp-in-bgaA_F2_Rev / D39_bgaA_Spec-cbpA-ins_Rev_1_seq | CCACCTACAATTCATAAGAATAC |
| PMR120 | D39_bgaA_Spec-ins_Fwd_2_seq | GACTTAGAGGAATTACTACCTG |
| PMR121 | D39_bgaA_Spec-cbpA-ins_Fwd_3_seq | AGCAAAGTTAGACGCAGCTTTTG |
| PMR122 | D39_bgaA_Spec-cbpA-ins_Fwd_4_seq | AAGAGGAAGCTAAGGAACCT |
| PMR123 | D39_bgaA_Spec-cbpA-ins_Fwd_5_seq | TAAACAGCAATGGCGCTATG |
| PMR124 | D39_bgaA_ins_Rev_1_seq | ACATCCTGATGGCTGGTATT |
| PMR125 | D39_bgaA_Spec-cbpA-ins_Rev_2_seq | CATAGCGCCATTGCTGTTTA |
| PMR126 | D39_bgaA_Spec-cbpA-ins_Rev_3_seq | GGTTCCTTAGCTTCCTCTTTTA |
| PMR127 | D39_bgaA_Spec-cbpA-ins_Rev_4_seq | CAAAAGCTGCGTCTAACTTTGCT |
| PMR128 | D39_bgaA_Spec-ins_Rev_5_seq | ATAATGGCTCTTCTCACATCA |
| PMR129 | D39_clpC-SPD2021_Fwd_seq | CACTCTTCCTAGGCATCATTA |
| PMR130 | D39_clpC_Rev_seq | TTGAGATTCACAAGCAGTTGC |
| R768 | TN ID 1A.2 | GGCCACGCGTCGACTAGTACNNNNNNNNNNNNNN |
| R760 | TN ID 1B (Binds to end of transposon) | GGACACGGGTAAAATCATACC |
| R761 | TN ID 2A (Binds to tail of random primer from first PCR) | GGCCACGCGTCGACTAGTAC |
| R762 | TN ID 2B (Binds to Tn-end, within product from first PCR) / Tn ID Sanger sequencing primer | GGAAATGCAGTGGCTGAATC |
| PMR44 | D39_lytB-F1_Fwd | ACTTTGTCAAAGACCCTG |
| PMR45 | D39_lytB-F1_in_Rev | gtaactgggcCCAAAATTCTCCTAAATTCATATTAC |
| PMR46 | D39_lytB-Franc_in_Fwd | agaattttggGCCCAGTTACCAAATACAG |
| PMR47 | D39_lytB-Franc_in_Rev | acttatagtaCCTGTCTTTGGGCAGATAAAG |
| PMR48 | D39_lytB-F2_in_Fwd | caaagacaggTACTATAAGTGAATATGATTTGAGTG |
| PMR49 | D39_lytB-F2_Rev | GTGCTTCTCGTTTCTCTTTAC |
| PMR50 | D39_lytB-F1_out_Rev | acttatagtaCCAAAATTCTCCTAAATTCATATTAC |
| PMR51 | D39_lytB-F2_out_Fwd | agaattttggTACTATAAGTGAATATGATTTGAGTG |
| PMR99 | D39_lytB_Fwd_seq | AGTCTTTCCAGTAGCCCTCAAA |
| PMR100 | D39_lytB_Rev_seq | TTCCATGCTAAGGACATTCCTG |

**References for supplementary material**

1. Bridges, A. A., Prentice, J. A., Fei, C., Wingreen, N. S. & Bassler, B. L. Quantitative input–output dynamics of a c-di-GMP signal transduction cascade in Vibrio cholerae. PLOS Biology **20**, e3001585 (2022).

2. Brissac, T. et al. Capsule Promotes Intracellular Survival and Vascular Endothelial Cell Translocation during Invasive Pneumococcal Disease. mBio **12**, 10.1128/mbio.02516-21 (2021).

3. Matela, A. M., Siatkowski, C. W., Yan, C., Thiagarajan, S. & Cooper, V. S. Student-led experimental evolution reveals novel biofilm regulators of adaptation to multiple niches. 2025.06.06.658356 Preprint at https://doi.org/10.1101/2025.06.06.658356 (2025).

4. Armbruster, C. R. et al. Heterogeneity in surface sensing suggests a division of labor in Pseudomonas aeruginosa populations. eLife **8**, e45084 (2019).

5. Mike, L. A. et al. A systematic analysis of hypermucoviscosity and capsule reveals distinct and overlapping genes that impact Klebsiella pneumoniae fitness. PLOS Pathogens **17**, e1009376 (2021).

6. Pariseau, D. A., Ring, B. E., Khadka, S. & Mike, L. A. Cultivation and Genomic DNA Extraction of Klebsiella pneumoniae. Current Protocols **4**, e932 (2024).
